# Draft genome sequence of subterranean clover, a reference for genus *Trifolium*

**DOI:** 10.1038/srep30358

**Published:** 2016-08-22

**Authors:** Hideki Hirakawa, Parwinder Kaur, Kenta Shirasawa, Phillip Nichols, Soichiro Nagano, Rudi Appels, William Erskine, Sachiko N. Isobe

**Affiliations:** 1 Kazusa DNA Research Institute, Kazusa-Kamatari 2-6-7, Kisarazu, Chiba 292-0818, Japan; 2 Centre for Plant Genetics and Breeding, The University of Western Australia, 35 Stirling Highway, Crawley, WA 6009, Australia; 3 Department of Agriculture and Food Western Australia, 3 Baron-Hay Court, South Perth, WA 6151, Australia; 4 School of Plant Biology, The University of Western Australia, 35 Stirling Highway, Crawley, WA 6009, Australia; 5 Murdoch University, 90 South Street, Murdoch, WA 6150, Australia

## Abstract

Clovers (genus *Trifolium*) are widely cultivated across the world as forage legumes and make a large contribution to livestock feed production and soil improvement. Subterranean clover (*T. subterraneum* L.) is well suited for genomic and genetic studies as a reference species in the *Trifolium* genus, because it is an annual with a simple genome structure (autogamous and diploid), unlike the other economically important perennial forage clovers, red clover (*T. pratense*) and white clover (*T. repens*). This report represents the first draft genome sequence of subterranean clover. The 471.8 Mb assembled sequence covers 85.4% of the subterranean clover genome and contains 42,706 genes. Eight pseudomolecules of 401.1 Mb in length were constructed, based on a linkage map consisting of 35,341 SNPs. The comparative genomic analysis revealed that different clover chromosomes showed different degrees of conservation with other Papilionoideae species. These results provide a reference for genetic and genomic analyses in the genus *Trifolium* and new insights into evolutionary divergence in Papilionoideae species.

The legume family (Leguminosae) is the third-largest plant family, consisting of 751 genera and ca. 19,500 species^1^, including several agronomically important crop and forage species. Among the forage legumes, alfalfa (*Medicago sativa* L.), white clover (*Trifolium repens* L.) and red clover (*T. pratense* L.) are the most economically important perennial pasture legumes and used in many temperate regions of the world. Of the annual forage legume species, subterranean clover (*T. subterraneum* L.) makes the greatest contribution globally to livestock feed production and soil improvement^1^, particularly in Australia, where it has been sown over 29 million ha and 45 cultivars have been registered since the early 1900s^2^. A further 11 annual clover species have been commercialized^3^.

Draft genome sequences are available in several legume species, including the crops soybean (*Glycine max* (L.) Merr.)^4^, common bean (*Phaseolus vulgaris* L.)^5^, chickpea (*Cicer arietinum* L)^6^ pigeon pea (*Cajanus cajan* (L.) Millsp.)^7^ and mungbean (*Vigna radiata* (L.) Wilezek)^8^, while among the forage legumes, draft sequences are available for red clover (*T. pratense* L.)^9^ and the model legume species, *Lotus japonicus* L.^10^ and *Medicago truncatula* Gaertn. (barrel medic)^11^. No draft genome sequences have been published for *M. sativa*, *T. repens* or any of the annual *Trifolium* species.

Subterranean clover has been proposed as a reference species for genetic and genomic studies within the genus *Trifolium*, as it is an annual, diploid (2n = 16), predominantly autogamous species with a relatively small genome size of 540 Mbp^2^ This contrasts with *T. pratense* and *T. repens*, which are allogamous perennials, with the latter also an allotetraploid, making genetic and genomic analyses difficult. Understanding the genetics of important traits in subterranean clover can provide a pathway to understanding the genetics in these more genetically complex species. The results from this study will provide a reference for genetic and genomic analyses in the *Trifolium* genus and also other forage and crop legumes.

This report presents the first draft genome sequence of an annual clover, subterranean clover. Eight pseudomolecules were constructed based on Illumina and Roche 454 assembled genome sequences and a high density SNP linkage map. For a detailed understanding of diversification among legume species, genome structures and gene characteristics were compared with four legume species in the subfamily Papilionoideae, common bean (*Phaseolus vulgaris*, v1.0)^5^ *L. japonicus,* (rel.3.0)^10^*, M. truncatula* (4.0 v1)^11^ and red clover (v2.1)^9^. Common bean is classified into the Millettioid clade (warm season legumes), whereas the other three species are in the Hologalegina clade (cool season legumes)^12^. *M. trunactula* and red clover belong to the same tribe, Trifolieae. Availability of a fully sequenced genome will be exploited for the analysis of genetic diversity and trait-dissection, as well as gene tagging for marker-assisted selection. Additionally, functional genomics, transcriptomics, and proteomics can be used more precisely and effectively in forage legume improvement.

## Results

### Genome sequencing and Assembly

The Australian subterranean clover variety, cv. Daliak, was subjected to whole genome shotgun sequencing. Single-end (SE), 520–660 bp paired-end (PE) and 2 kb, 5 kb, 8 kb, 10 kb, 15 kb and 20 kb mate-pair (MP) libraries were constructed for Roche 454 GS FLX+, Illumina MiSeq and HiSeq 2000 platforms, respectively (Supplementary Table S1). A total of 6.8 Gb overlap fragment (OF) reads were created from 16.7 Gb MiSeq PE reads by COPE^13^ (Supplementary Fig. S1). Together with the 2.8 Gb 454 reads, the 6.8 Gb OF reads were assembled by Newbler 2.7. The resultant number of contigs was 101,010, totaling 414.8 Mb in length (Supplementary Table S2). In parallel, 23.2 Gb HiSeq SE and PE reads were assembled by SOAPdenovo2^14^ (kmer = 61) and GapCloser 1.10 (*p* = 31). The assembled 603,937 sequences, totaling 421.8 Mb in length, were merged with the 101,010 Newbler contigs by GAM-NGS^15^ and 92,729 merged scaffolds were consequently generated. A total of 44,900 super-scaffolds were created by further scaffolding of the 92,729 sequences using SSPACE2.0^16^, giving a total of 125.3 Gb MP reads. After excluding contaminated sequences and those shorter than 300 bases, the remaining 27,228 sequences were subjected to subsequent analysis as a draft genome sequence, TSUd_r1.0 (Supplementary Table S2).

### SNP map and pseudomolecule construction

**Figure 1:**
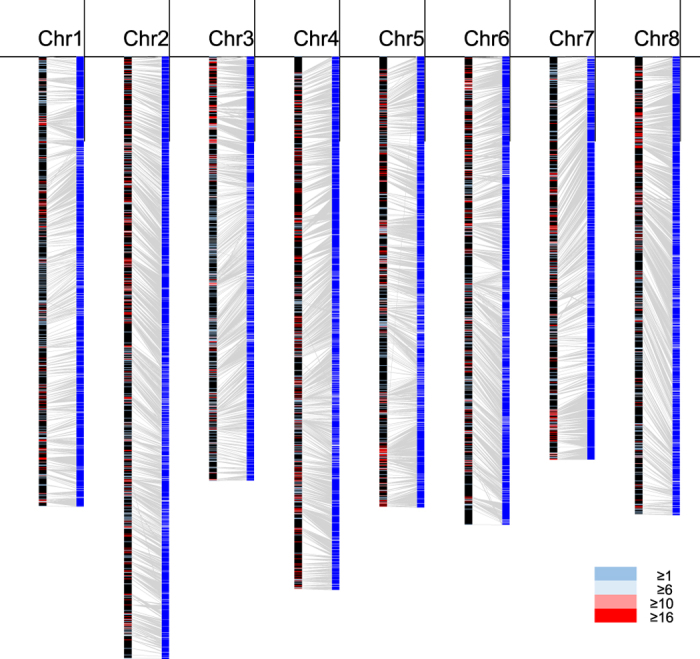
Anchoring the subterranean clover genome assembly to the genetic linkage map. The 92S80 SNP linkage map (left bars) and 1,702 anchored TSUd_r1.1 scaffolds (right bars) with 35,341 SNPs. The crossbars on the linkage map show the position of SNP bins. The colors of bars represent numbers of mapped SNPs on each bin.

A SNP linkage map was constructed with the F_2_ population 92S80, generated from a cross between the subterranean clover cultivars Woogenellup and Daliak^17^. A total of 899,064 candidate SNPs were identified by mapping the 25.8 Gb Woogenellup and 337.9 Gb F_2_ HiSeq 1000 PE reads onto the TSUd_r1.0 sequences, and 52,635 SNPs were subsequently selected for Axiom^®^ myDesign^TM^ TG Array. Based on the genotypes of the 155 F_2_ progenies obtained from the array, a SNP linkage map was constructed with 35,341 SNPs (Supplementary Table S3 and http://clovergarden.jp/). The mapped SNPs generated a total of 2,153 bins that were randomly located on eight linkage groups totaling 2,084 cM in length (Fig. 1, Supplementary Table S3).

A total of 1,505 TSUd_r1.0 scaffolds were anchored onto the linkage map through the mapped SNPs. Of the anchored scaffolds, 158 were located in multiple positions that suggested mis-assembly. Hence, the 158 scaffolds were split into 355 scaffolds according to the SNP positions on the linkage map. In addition, reverse complement sequences were created as revised scaffold sequences when the original sequences were anchored on the linkage map in a reverse direction. The modified TSUd_r1.0 sequences were designated as TSUd_r1.1. TSUd_r1.1 consisted of 27,424 scaffolds with 471,834,188 bp including 57,776,905 N bases. GC% and N50 were 33.3% and 287,605 bp, respectively (Table 1, Supplementary Table S4). The assembled sequences covered 85.4% of the subterranean clover genome (552.4 Mb), which was estimated based on kmer frequency distribution (kmer = 17, Supplementary Fig. S2). Assembly quality of the TSUd_r1.1 sequences was investigated by mapping assembled sequences onto the 248 core eukaryotic genes (CEGs) by CEGMA^18^. The numbers of ‘complete’ and ‘complete or partial’ mapped CEGs were 237 (95.6%) and 243 (98.0%), respectively, indicating high reliability of the assembled sequences.

The total number and length of anchored TSUd_r1.1 scaffolds on the SNP map were 1,702 and 384,208,136 bp (81.4% of TSUd_r1.1), respectively. Eight pseudomolecules, chr1-chr8, were constructed by connecting the anchored TSUd_r1.1 scaffolds with insertions of 10,000 N bases in between adjacent scaffolds (Fig. 1 and Table 1). Total length of the eight pseudomolecules was 401.1 Mb including 54.9 Mb Ns. The length of each pseudomolecule ranged from 42.7 Mb (Chr7) to 63.7 Mb (Chr2, Supplementary Table S4).

### Repetitive sequences

Repetitive sequences in the assembled genome were identified by RepeatScout^19^ and RepeatMasker. The total length of repetitive sequences in TSUd_r1.1 was 216.8 Mb, including 72.9 Mb known types and 143.9 Mb unique repeats (Supplementary Table S5). Of the known types of repeats, Class I LTR (Long Terminal Repeat) elements were observed most frequently. The ratios of repetitive sequences of the eight pseudomolecules ranged from 36.1% (chr7) to 46.0% Mb (chr6). A higher ratio of repetitive sequences was observed in chr0 (scaffolds that were not in pseudomolecules). Of the unique repeats observed on the eight pseudomolecules, 67.4Mb (62.8%) sequences were commonly observed in at least one of the other four legume species (red clover, *M. truncatula*, *L. japonicus* and common bean), while the other 39.8 Mb (37.2%) were subterranean clover-specific (Supplementary Table S6). A total of 61,402 SSR sequences were identified in TSUd_r1.1 (Supplementary Table S7). The average frequency of SSRs in overall and coding sequence (CDS) was 12.4 and 5.1 per 100 kb, respectively. The SSR frequency in CDS was lower than in the other four legume species.

### Gene prediction and annotation

**Figure 2:**
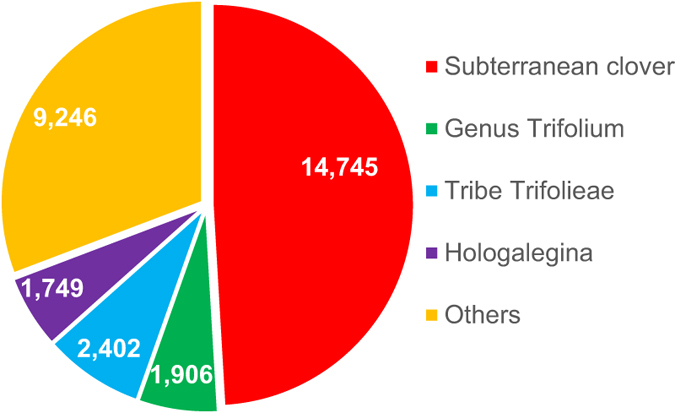
Number of subterranean clover protein encoding gene clusters with other legume species. Subterranean clover: subterranean clover (S)-specific genes, Genus *Trifolium*: S- red clover (R) cluster, Tribe Trifolieae: S*-M. truncatula* (M) and S-R-M clusters, Hologalegina: S*-L. japonicus* (L), S-L-R, S-M-L and S-M-L-R, gene clusters, Others: S- common bean (C), S-C-R, S-M-C, S-L-C, S-R-M-C, S-R-L-C, S-M-L-C and S-R-M-L-C clusters.

Gene prediction of TSUd_r1.1, employing MAKER^20^, yielded a total 28,372 genes. Moreover, a further 14,334 genes were additionally predicted by Augustus^21^, giving a total of 42,706 predicted genes with average coding sequence length of 1,123 bp (Supplementary Table S8). In addition, 1,007 tRNA and 92 rRNA encoding genes were identified (Supplementary Table S9). Among the 42,706 putative genes, 37,085 (86.8%) were classified as non-TE genes whereas 5,621 (13.2%) were transposon elements (TEs), based on BLAST and domain searches against NCBI NR and InterPro^22^ databases, respectively. For comparisons with the gene sequences in other legume species, 36,800 subterranean clover putative non-TE genes were clustered with the genes predicted in red clover, *M. truncatula*, *L. japonicus*, and common bean. This generated a total of 30,048 clusters. The number of subterranean clover-specific clusters was 14,745, whereas that commonly observed in genus *Trifolium* (subterranean and red clovers), tribe Trifolieae (clovers and *M. truncatula*) and clade Hologalegina (clovers, *M. truncatula* and *L. japonicus*) was 1,906, 2,402 and 1,749, respectively (Fig. 2). A total of 6,388 (21.3%) clusters were observed in all five legume species (Supplementary Fig. S3).

The 36,800 putative genes were further annotated using GO^23^, KOG^24^ and KEGG^25^ databases. A total of 30,543 (83.0%) genes were annotated with GO categories, including 10,328 genes involved in biological processes, 4,400 genes coding for cellular components and 15,815 genes associated with molecular function (Supplementary Fig. S4). Of the 16,995 subterranean clover-specific genes (See Fig. 2), only 6,449 (38%) genes were annotated while most genes (12,351, 88%) in the ‘Other’ cluster were classified in the GO categories. This result indicates that many of the subterranean clover-specific genes are novel with unknown function. Meanwhile, a total of 31,335 putative genes showed significant similarity to genes in the KOG database and 3,520 (11.2%), 6,004 (19.2%) and 5,165 (16.5%) genes were annotated in ‘information storage and processing’, ‘cellular processes and signaling’ and ‘metabolism’, respectively (Supplementary Fig. S5). Genes belonging to the tribe Trifoliae cluster showed a higher ratio of ‘poorly characterized’ genes. The putative genes were also mapped onto a total of 1,688 enzyme encoding genes on KEGG pathways categorized ‘1. Metabolism’ (Supplementary Table S10).

### Whole structure and variance in the genome of subterranean clover

**Figure 3:**
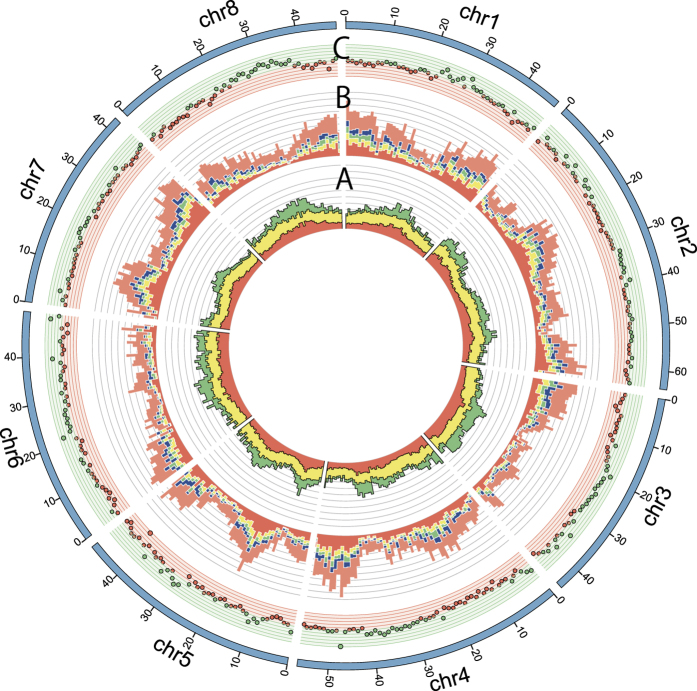
Graphical view of the subterranean clover genome structure. (**A**) Ratios of repetitive sequences in 1 Mb window. Red bars represent known repeats. Yellow bars show unique repeats commonly observed in the legume species, red clover*, M. truncatula*, *L. japonicus* and common bean, while green bars represent subterranean clover-specific sequences. The distance between horizontal lines shows 10% (**B**) Numbers of putative subterranean clover genes in a 1 Mb window. Red bars represents subterranean clover genes clustered with each of the other four legume species (red clover*, M. truncatula*, *L. japonicus* and common bean), yellow bars with three of the four species, green bars with two of the four species, and blue bars with only one of the species, while orange bars show subterranean clover-specific genes. The distance between horizontal lines represents 20 genes. (**C**) CNV distribution. Green and red dots show log2 ratio plus and minus values, respectively. The distance between horizontal lines represent log2 ratio of 0.1.

When the distribution of repetitive and putative gene sequences were surveyed at a whole genome scale, larger ratios of repetitive sequences and a smaller number of genes were observed in midsections of chr1, chr3, chr4, chr6, chr7 and chr8, suggesting heterochromatin regions (Fig. 3A). However, candidate heterochromatin regions in chr2 and chr5 were insufficiently resolved to observe clearly. The ratios of common genes among the five legume species were lower in chr3, chr6 and chr8 than the other pseudomolecules (Fig. 3B). Higher ratios of subterranean clover-specific genes tended to be observed in euchromatin regions, with gene density being higher in chr1 and chr7 and lower in chr3 and chr6.

Copy number variations (CNVs) in the genomes of ‘Daliak’ and ‘Woogenellup’ were detected using the CNV-seq program^26^. Higher log2 ratio (log-transformed ratio of the number of mapped reads in Woogenellup to the number in Daliak) were observed in the regions where densities of repetitive sequences were high (Fig. 3C). The functions of the 35,341 SNPs mapped on the pseudomolecule on gene functions were predicted using SnpEff 27, which groups SNPs into four categories (high-impact, moderate, modifier, and low), based on their positions in the genome sequences (Supplementary Fig. S6). Among 36,723 annotations on the 35,341 SNPs, 287 (0.8%) were classified as high-impact SNPs, including “splice acceptor and donor variants”, “loss of the start codon”, or “gain/loss of the stop codon”. Another 4,894 loci (13.3%) were classified as “moderate effects” (mis-sense variants), 26,107 (71.1%) as “modifiers” (e.g. variants in intergenic regions and introns), and 5,435 (14.8%) as “low-impact” (e.g. synonymous variants, Supplementary Table S11).

### Comparative analysis with other legume species

**Figure 4:**
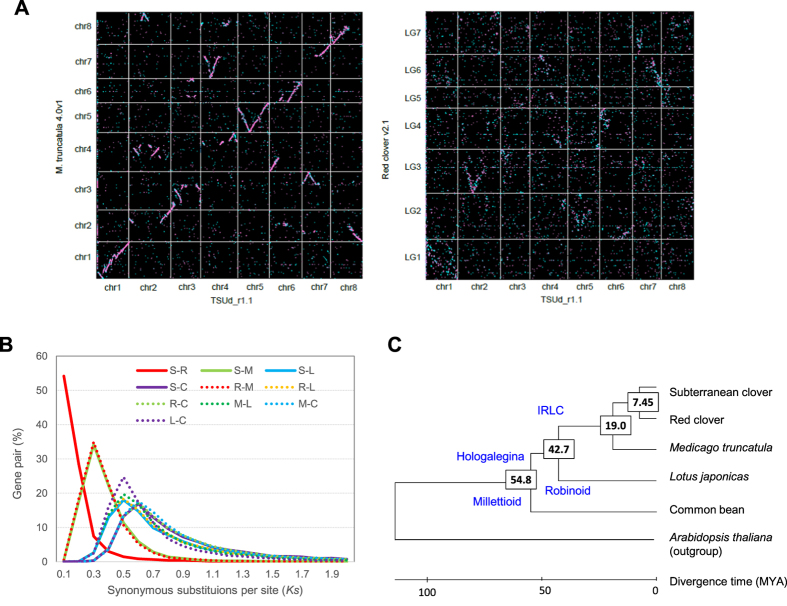
Comparative analysis with other legume species. (**A**) Graphical view of syntenic relationships between subterranean clover and *M*. *truncatula* (left) and between subterranean clover and red clover (right). Pink and blue dots show homologous sequences of Tsud_r1.1 with forward and reverse directions against the reference sequences. (**B**) Distribution of Ks values of orthologous gene pairs in subterranean clover and the four legume species. Subterranean clover, red clover, *M. truncatula*, *L. japonicus* and common bean are abbreviated to S, R, M, L and C, respectively. (**C**) A phylogenetic tree of 280 common single copy genes of the five legume species and *A. thaliana*.

A similarity search was performed, based on comparisons with the translated protein sequences of red clover, *M. truncatula, L. japonicus* and common bean (Fig. 4A and Supplementary Fig. S7). Alignment of homologous sequence pairs along each pseudomolecule (Tsud_chr) revealed obvious syntenic relationships with chromosomes in *M. truncatula* (Mt_chr). Single synteny blocks were observed for three pseudomolecules (Tsud_chr1-*M. truncatula* chromosome 1(Mt_chr1), Tsud_chr3-Mt_chr3 and Tsud_chr5-Mt_chr5). Tsud_chr, 6, 7 and 8 shared two synteny blocks with Mt_chr3-chr6, Mt_chr3-chr6 and Mt_chr2-Chr8, respectively. The remaining two pseudomolecules shared three synteny blocks with Mt-chrs. Duplicated synteny blocks were observed in Tsud_chr2, chr4, chr5 and chr6. By contrast, clear synteny blocks were not shown between subterranean and red clovers. This might be caused by the shorter pseudomolecule (164.2 Mb of 309 Mb assembled genome) and larger ratio of unknown amino acid sequences in red clover (Percentages of X in translated protein sequences was 1.48% in red clover and 0.02% in subterranean clover). Nevertheless, possible synteny blocks were observed between Tsud_chr1-red clover linkage group 1 (Rc_LG1), Tsud_chr2-Rc_LG3, Tsud_chr5-Rc_LG2, Tsud_chr6-RC_LG2-LG4 and Tsud_chr7-RC_LG6-LG7. Clear synteny blocks were also observed between the subterranean clover genome and the other legume species. However, the blocks were more fragmented when compared with both *L. japonicus* and common bean, than when compared against *M. truncatula.* Overall, Tsud_chr1 is more conserved across the five legume species than the other pseudomolecules.

Fifty-four percent of gene pairs between subterranean and red clovers showed a synonymous-substitution rate (*Ks* value) less than 0.1, suggesting a close relationship between the two clovers (Fig. 4B). A similar distribution of *Ks* value was observed between *M. truncatula* and the two clovers, although the basic chromosome numbers of the two clovers differ (subterranean clover n = 8, red clover n = 7). Interestingly, the peaks of *Ks* value between *L. japonicus* and common bean were almost the same as that between the three tribe Trifoleae species and *L. japonicus*.

To infer the divergence time among *Arabidopsis thaliana* and the five legume species (common bean, *L. japonicus*, *M. truncatula*, red clover and subterranean clover), a phylogenetic tree was constructed based on 280 single copy genes commonly observed across the six species. According to the phylogenetic tree, it was estimated that the Hologalegina and Millettioid clades diverged ~54.8 MYA (Fig. 4C, Supplementary Fig. S8), similar to the study of Lavin *et al.*^28^. The divergence time between Robinioid (including *L japonicus*) and IRLC (Inverted Repeat Lacking Clade, including tribe Trifoliae) clades was ~42.7 MYA, while the genera *Trifolium* and *Medicago* diverged ~19.0 MYA. These two time estimations were more recent than the previous study of ~47.6 MYA and ~23 MYA, respectively^9^, which could be due to the different number of single copy genes used in the studies (280 in this manuscript and 818 in de Vega *et al.*^9^). The divergence time between subterranean clover and red clover was estimated as 7.45 MYA.

## Discussion

We constructed a 471.8 Mb subterranean clover draft genome, including eight pseudomolecules, totaling 401.1 Mb. This is the first draft genome of an annual forage clover. The assembled sequence covers 85.4% of the subterranean clover genome and is of high quality, according to CEGMA. Based on the 42,706 predicted genes, 14,745 subterranean clover-specific gene clusters were generated. These gene clusters will be a source for discovery of unique and potentially valuable genes in the species for a range of traits, including those leading to increased biomass production and persistence, disease and pest resistance, increased phosphorous-use efficiency and reduced methane emissions from grazing ruminant livestock^2^. Moreover, the high density linkage map, consisting of 35,341 SNPs and 287 high-impact SNPs annotated by SnpEff, will also greatly advance genetic and genomic analyses of subterranean clover.

Comparative analysis with the other legume species provided an insight into the evolution of Papilionoideae species. Synteny analysis revealed that the whole chromosome structure of chr1 in subterranean clover was highly conserved across the five Papilionoideae species compared and markedly more conserved than the other chromosomes. By contrast, duplicate synteny blocks were observed in subterranean clover chr2 against *M. truncatula* and red clover, suggesting that the genome duplication in chr2 occurred after the divergence of red and subterranean clovers ~7.45 MYA.

It is difficult to obtain high quality draft genomes in many of the most important forage legume species. The perennial species, red clover, white clover and alfalfa, show strong self-incompatibility, making it difficult to produce homozygous plants for genetic and genomic analysis. Even though a draft genome for red clover has been published^9^, the quality of the subterranean clover genome in this study is much higher. For example, the percentages of complete and partial mapped CEGs by CEGMA in red clover were 85.5% and 95.6%, respectively, whereas those in subterranean clover were 95.6% and 98.0%. In addition, white clover and alfalfa are polyploid species. Subterranean clover is much better suited than these species for genomic and genetic studies because it is an annual, autogamous and diploid species. the results obtained in this study are expected to be a reference for genetic and genomic analyses within the *Trifolium* genus and also among other forage legumes. In the case of subterranean clover, a genome scaffold, a high resolution QTL map, the development of a world core collection and extensive phenotyping for important agro-morphological and economic traits have generated sufficient molecular genetic information to establish a comprehensive molecular breeding platform.

## Materials and Methods

### Genome sequencing and Assembly

An Australian subterranean clover variety, cv. Daliak, was subjected to whole-genome shotgun sequencing using the Roche 454 GS FLX+, Illumina HiSeq 2000 and MiSeq platforms. Total cellular DNA was used for construction of a SE library for Roche 454 and PE libraries for Illumina HiSeq 2000 and MiSeq sequencing platforms according to the manufacturer’s instructions. A modified protocol proposed by Nieuwerburgh *et al.*^29^ was employed for MP library preparation. The obtained reads were subjected to quality control, as follows. Bases with quality scores less than 10 were filtered by PRINSEQ 0.20.4^30^ and adaptor sequences in the reads were trimmed using fastx_clipper from the FASTX-Toolkit 0.0.13 (http://hannonlab.cshl.edu/fastx_toolkit).

The 454 reads and OF reads created from MiSeq PE reads by COPE^13^ were assembled using Newbler 2.7 (Roche Diagnostics, IN, USA). In parallel, HiSeq PE reads were assembled by SOAPdenovo2 (kmer = 61)^14^ and the gaps closed by GapCloser 1.10 (p = 31) (http://soap.genomics.org.cn/soapdenovo.html). The generated scaffolds were merged with the Newbler contigs by GAM-NGS^15^. The resultant sequences were further subjected to scaffolding with the MP reads using SSPACE2.0 with the parameters (-k 3 -x 0)^16^. Potential contaminated sequences on the assembled scaffolds were identified and removed using BLASTN searches against the chloroplast genome sequence of *A. thaliana* (Accession number: NC_000932.1), mitochondrial genome sequence of *A. thaliana* (Accession number: NC_001284.2), bacterial genome sequences registered in NCBI (http://www.ncbi.nlm.nih.gov), and vector sequences from UniVec (http://www.ncbi.nlm.nih.gov/tools/vecscreen/univec/) with E-value cutoff of 1E-10 and length coverage ≥10%. Assembly quality was investigated by CEGMA^18^. Repetitive sequences were searched using RepeatScount 1.0.5^19^ and RepeatMasker 3.30 (http://www.repeatmasker.org). SSR motifs were identified using the SciRoKo software^31^ in the “MISA” mode with default parameters. The genome size of subterranean clover was estimated by kmer frequency distribution (kmer = 17) using Jellyfish ver. 2.1.1^32^.

### SNP map and pseudomolecule construction

A total of 155 F_2_ derived F_4_ bulked DNAs of the 92S80 population were used for SNP linkage map construction. The trimmed Illumina SE and PE reads of the Woogenellup parent (DRR018261, DRR018262, DRR024180 and DRR024181) were mapped onto TSUd_r1.0 using Bowtie 2 2.2.34 (parameters maxins = 1000)^33^. SNP candidates were called based on the mapping result using samtools mpileup ver.1.1.19 (parameters: –Duf –d 1000000)^34^, and subsequently filtered using VCFtools ver 1.1.19^35^. An Axiom^®^ myDesign^TM^ TG Array was designed for a total of 59,105 SNPs, which exhibited Minor allele frequency ≥30 and Missing data ≤30%. The linkage map was constructed using MultiPoint 3.3 (http://www.multiqtl.com/) with the following parameters: Population type = F2, Min LOD threshold = 10.0, Max threshold rf = 0.25, Kosambi mapping function.

### Gene prediction and annotation

tRNA genes were predicted using tRNAscan-SE ver. 1.23^36^ with default parameters. rRNA genes were predicted by BLAST searches with an E-value cutoff of 1E-10. The *A. thaliana* 5.8S and 25S rRNAs (accession number: X52320.1) and 18S rRNA (accession number: X16077.1) were used as query sequences.

Protein-encoding sequences in the assembled genomic sequences were predicted by MAKER^20^. Published SRA transcript sequences of red clover (SRX351919, SRX351918, SRX351917, SRX351791) and white clover (ERX324290) were assembled by Trinity^37^ for evidence-based gene prediction. The assembled 431,700 red clover and 217,133 white clover unigenes were mapped onto the subterranean pseudomolecules by BLAT (-t = dnax –q = dnax minIdentity = 25), together with the 62,319 *M. truncatula* CDS (4.0 v1). In parallel, *Ab initio* gene prediction was performed by Augustus^21^ using the *A. thaliana* training set. The two sets of predicted gene sequences were merged by MAKER. Genes related to transposable elements (TEs) were detected by BLASTP searches against the NCBI non-redundant (nr) protein database (http://www.ncbi.nlm.nih.gov) with an E-value cutoff of 1E-10, and InterProScan^38^ searches against the InterPro database^22^ with an E-value cutoff of 1.0. The putative non-TE genes were classified into the plant gene ontology (GO) slim categories^23^, and the “euKaryotic clusters of Orthologous Groups” (KOG) categories^24^ and then mapped onto the Kyoto Encyclopedia of Genes and Genomes (KEGG) reference pathways^25^.

### SNP annotation and CNV analysis

CNVs were analyzed using CNV-seq ver. 0.2.7 (parameters: –genome-size 239,146,348)^26^. SNP effects on gene function in TSUd_r1.1 were predicted using SnpEff ver. 4.0 g (parameters: –no-downstream, -no-upstream)^27^.

### Comparative analysis

Translated protein sequences of subterranean clover were clustered by using the CD-HIT program^39^ with those of common bean (v1.0)^5^, *L. japonicus* (rel.3.0)^10^, *M. truncatula* (4.0 v1)^11^, and red clover (v2.1)^9^ with the parameters c = 0.6 and aL = 0.5. Homologous translated proteins sequences were searched by BLAST searches with an E-value cut-off of 1E-100, and the synteny plot was made using the gnuplot program (http://www.gnuplot.info). *Ks* value was estimated for the gene pairs with reciprocal best hits of BLAST searches with an E-value cut-off of 1E-10, using KaKs Calculator^40^.

### Phylogenetic analysis

CD-HIT analysis against the six species (*A. thaliana* (TAIR10), common bean (v1.0), *L. japonicus* (rel.3.0), *M. truncatula* (4.0 v1), red clover (v2.1), and subterranean clover (TSUd_r1.0)) were conducted with the parameters c = 0.6 and aL = 0.9. The single copy genes in each cluster commonly conserved among the six species were applied to multiple alignment by MUSCLE 3.8.31^41^. The indels in the aligned sequences of the single copy genes were eliminated by Gblocks 0.91b^42^. The sequences of conserved blocks in the single copy genes were concatenated for each species and were used for construction of a phylogenetic tree by the Maximum-Likelihood (ML) algorithm, using MEGA 7.0.9 beta^43^ with the Jones-Taylor-Thornton (JJT) model as substitution model. The time values were calculated according to the distances in the phylogenetic tree constructed by Maximum Likelihood method. The uniform rates defined in the MEGA program were used for converting the distances to time values. In this step, *A. thaliana* was selected as outgroup. According to TIMETREE (http://www.timetree.org), the divergence time between *A. thaliana* and *M. truncatula* is 114.0 MYA, and this value was used for calibration. The divergence times among the six species were calculated by using MEGA 7.0.9 beta.

### Data availability

The genome assembly data (scaffolds and pseudomolecule), annotations, gene models, and SNPs on the linkage map are available at the Clover GARDEN (http://clovergarden.jp/). The genome sequence reads obtained are available from the DDBJ Sequence Read Archive (DRA) under the accession numbers DRA003274. The BioProject accession number of the study is PRJDB2012. The WGS and CON accession numbers of assembled sequences are BCLP01000001-BCLP01066167 (66,167 entries) and DF973112-DF976994 (3,883 entries). The protein IDs are GAU09980.1-GAU52038.1.

## Additional Information

**How to cite this article**: Hirakawa, H. *et al.* Draft genome sequence of subterranean clover, a reference for genus *Trifolium*. *Sci. Rep.* **6**, 30358; doi: 10.1038/srep30358 (2016).

## Supplementary Material

Supplementary Information

## Figures and Tables

**Table 1:** Statistics of the subterranean clover genome assembly.

	TSUd_r1.1	Pseudomolecules
Number of Sequences	27,424	8
Total length of sequences (bases)	471,834,188	401,148,136
Maximum length (bases)	2,878,652	63,731,624
Minimum length (bases)	300	42,658,284
Average length (bases)	17,205	50,143,517
N50 length (bases)	287,605	—
GC%	33.3	33.0
