# Supplementary material for: Draft genome sequence of subterranean clover, a reference for genus Trifolium
Source: Sci Rep. 2016 Aug 22;6:30358. doi: 10.1038/srep30358 (PMC4992838; doi:10.1038/srep30358)
Supplement: Supplementary Information [file srep30358-s1.pdf]

Draft genome sequence of subterranean clover, a reference for genus *Trifolium*

Hideki Hirakawa[1,6], Parwinder Kaur[2,6], Kenta Shirasawa[1], Phillip Nichols[3,4], Soichiro Nagano[1], Rudi Appels[5], William Erskine[2], Sachiko N. Isobe[1]*

[1]Kazusa DNA Research Institute, Kazusa-Kamatari 2-6-7, Kisarazu, Chiba 292-0818, Japan
[2]Centre for Plant Genetics and Breeding, The University of Western Australia, 35 Stirling Highway, Crawley, WA 6009, Australia
[3]Department of Agriculture and Food Western Australia, 3 Baron-Hay Court, South Perth, WA 6151, Australia
[4]School of Plant Biology, The University of Western Australia, 35 Stirling Highway, Crawley, WA 6009, Australia
[5]Murdoch University, 90 South Street, Murdoch, WA 6150, Australia
[6] Contributed equally

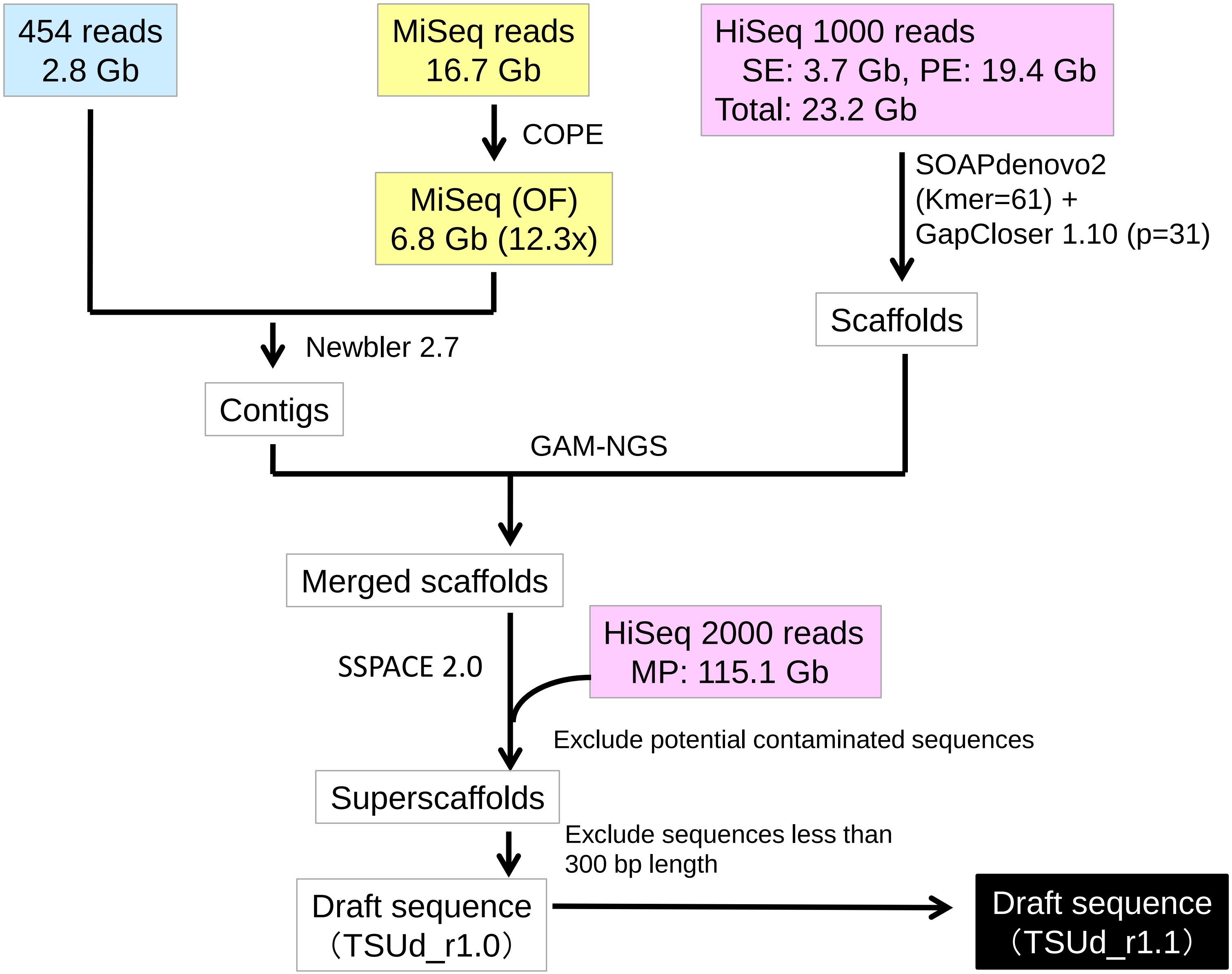

Supplementary Fig. S1. The strategy and status of sequencing and assembly of TSUd_r1.1. SE, PE, OF and MP represent single-end, paired-end, overlap fragments and mate-pair reads, respectively.

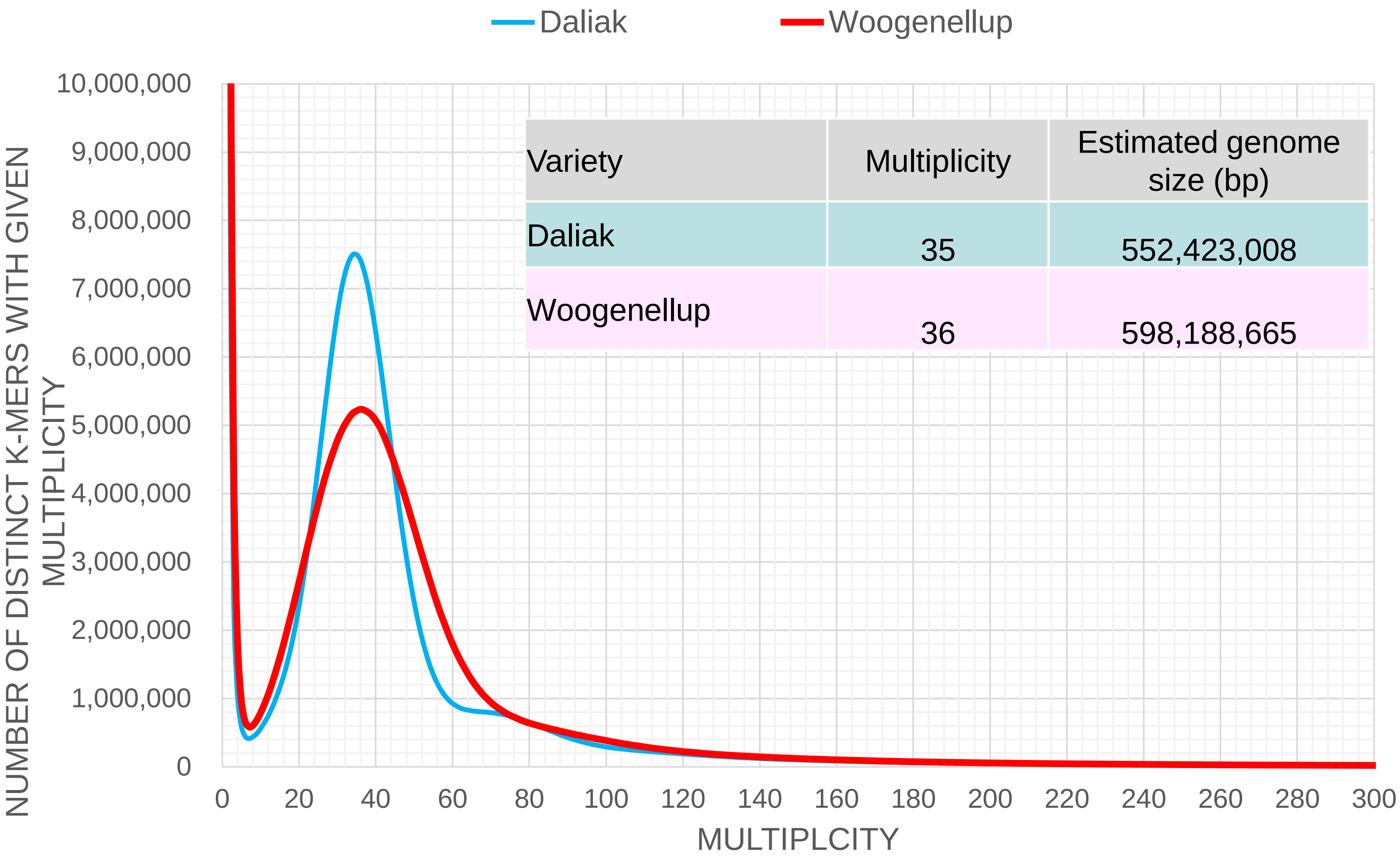

Supplementary Fig. S2. Genome size estimation with the distribution of the number of distinct kmers (kmer = 17) with the given multiplicity values.

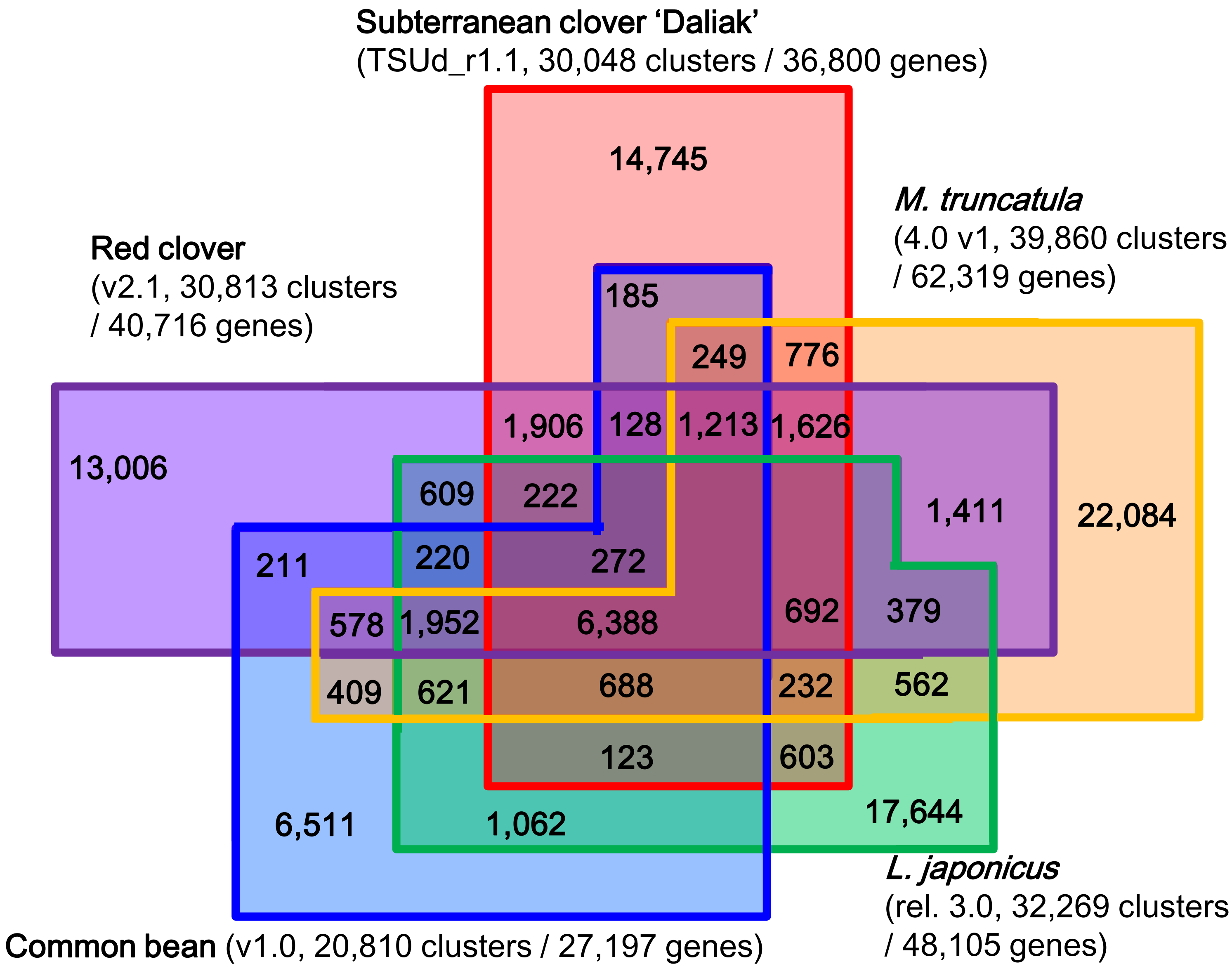

Supplementary Fig. S3. Venn diagram showing numbers of gene clusters in subterranean clover and the four legume species, red clover, *M. truncatula*, *L. japonicus* and common bean.

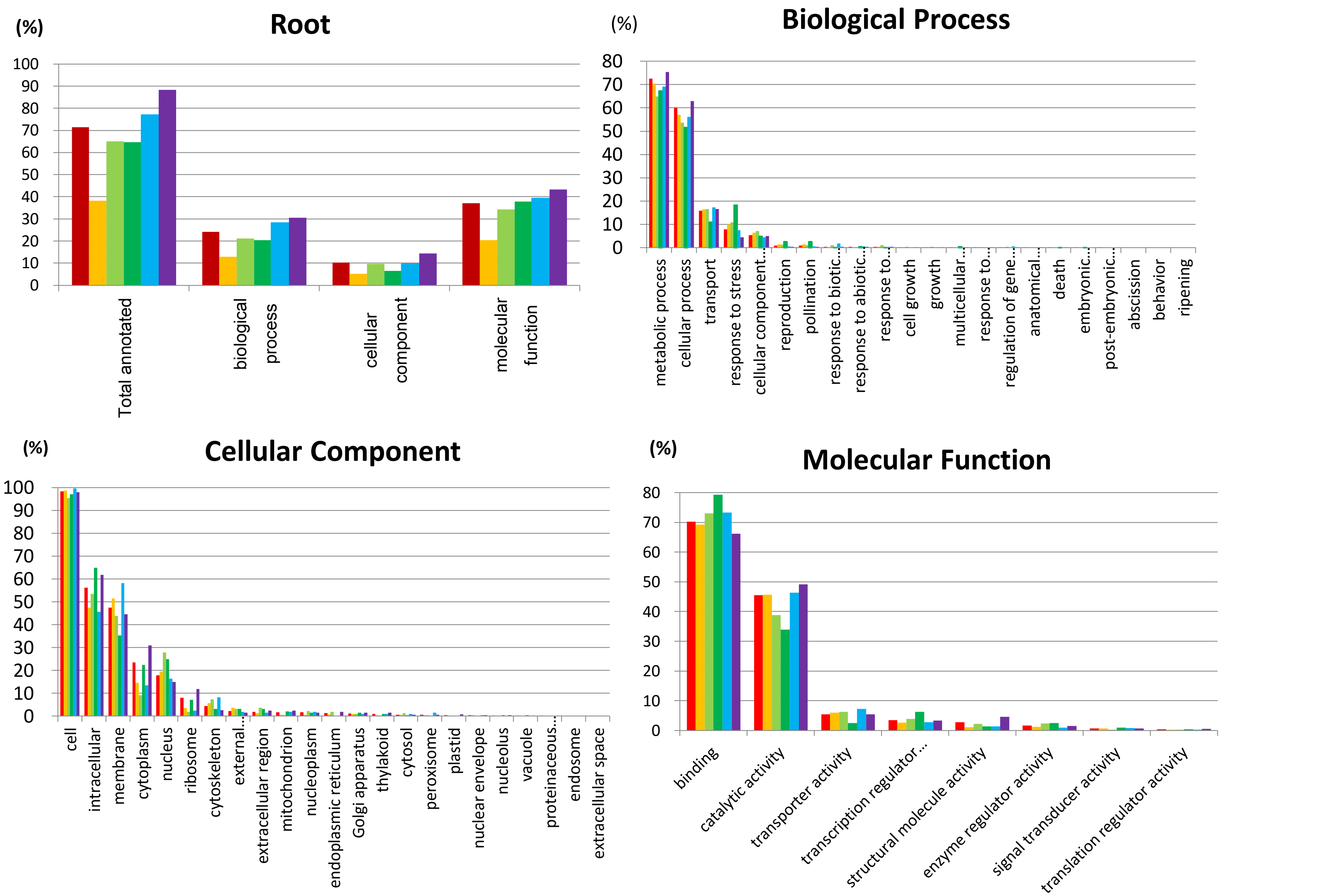

Supplementary Fig. S4. Classification of GO categories. Y axis represents percentages to total number of genes. Red: Tsud_r1.1 (36,800 genes), Yellow: Subterranean clover cluster (16,995 genes), Light green: Genus *Trifolium* cluster (2,283 genes), Green: Tribe Trifoliae cluster (3,010 genes), Light blue: Hologalegina cluster (2,161 genes), Purple: Other cluster (12,351 genes). For details of the clusters, see the Figure 2 legend.

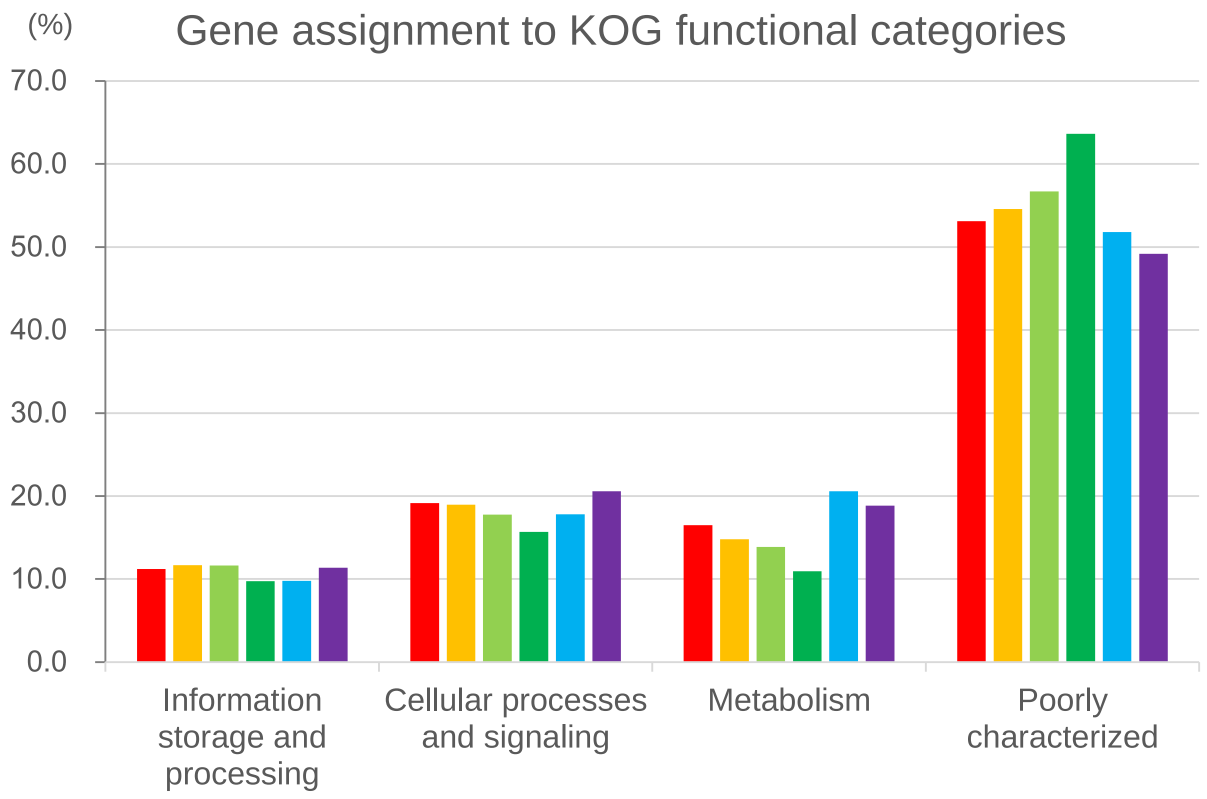

Supplementary Fig. S5. Gene assignment to KOG functional categories in subterranean clover. Y axis represents percentages to total number of genes. Red: Tsud_r1.1 (31,335 genes), Yellow: Subterranean clover cluster (10,966 genes), Light green: Genus Trifolium cluster (2,123 genes), Green: tribe Trifoliae cluster (2,925 genes), Light blue: Hologalegina cluster (2,279 genes), Purple: Other cluster (13,042 genes). For details of the clusters, see the Figure 2 legend.

Supplementary Figure S5 cont.

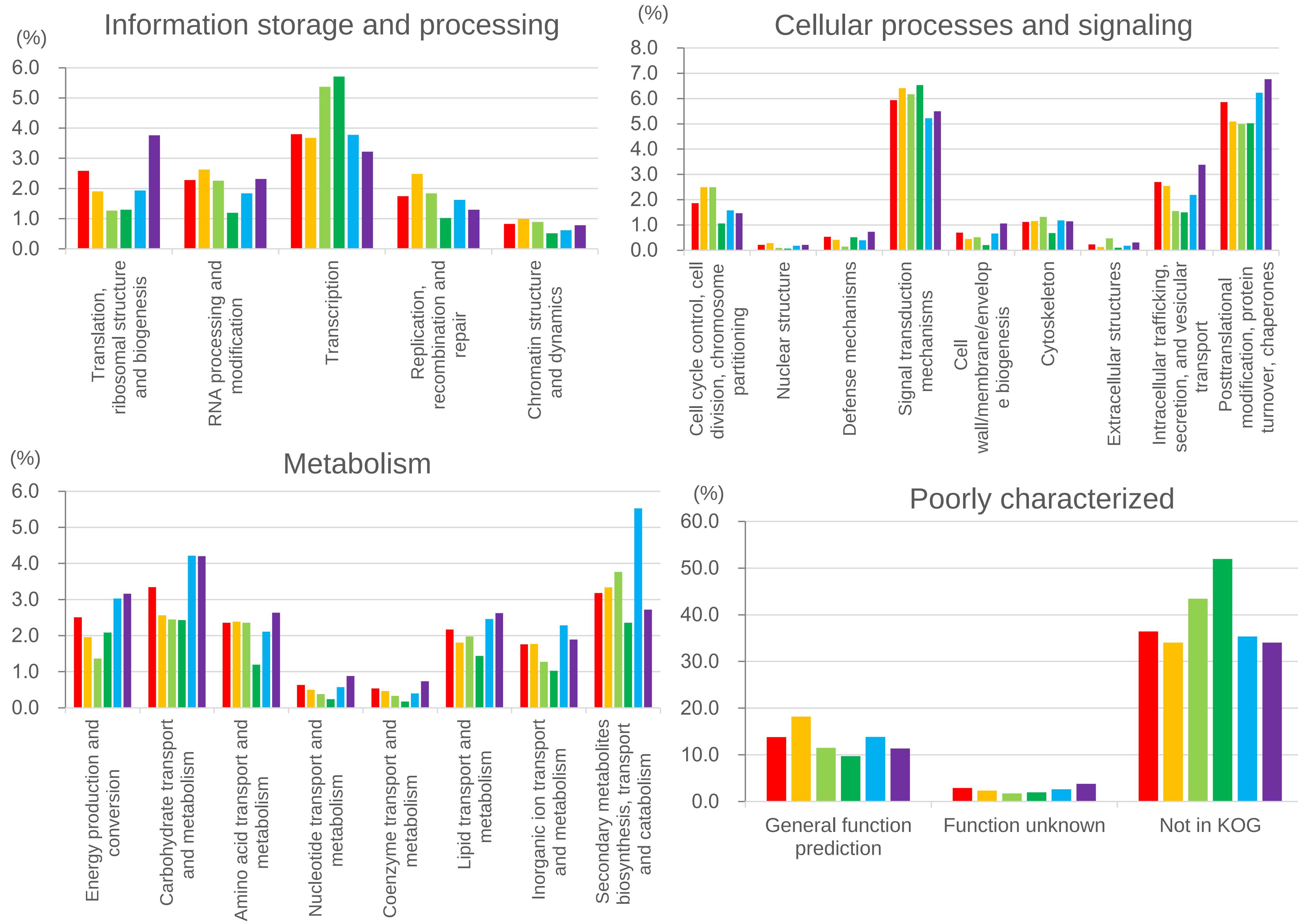

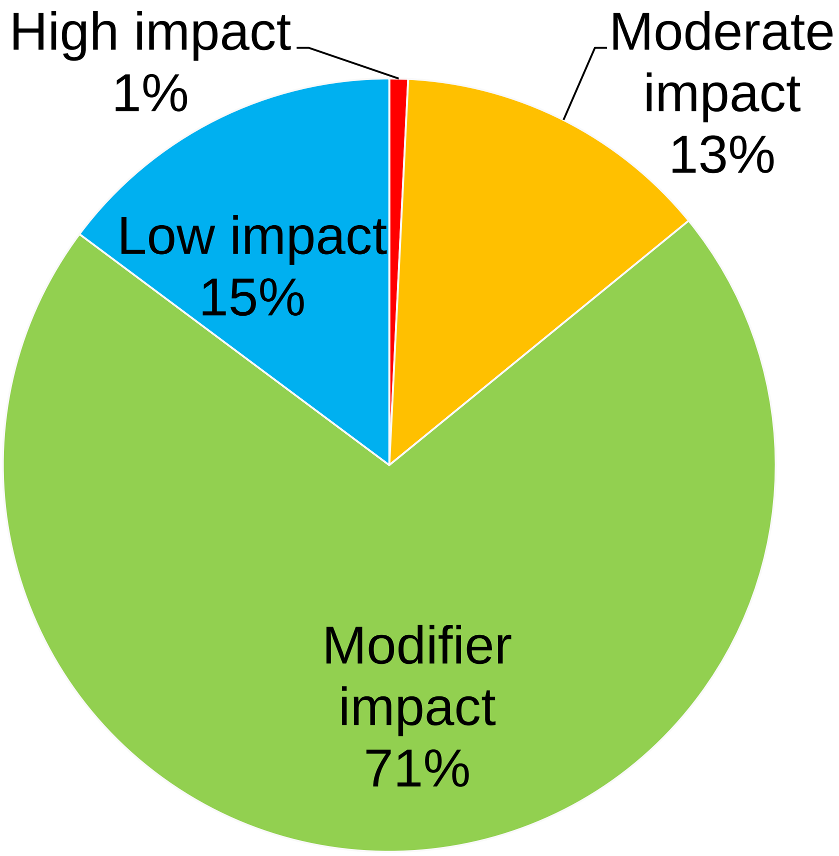

Supplementary Fig. S6. Effects of SNPs on gene functions that predicted by SnpEff program. Total number of the SNPs is 1,461,173.

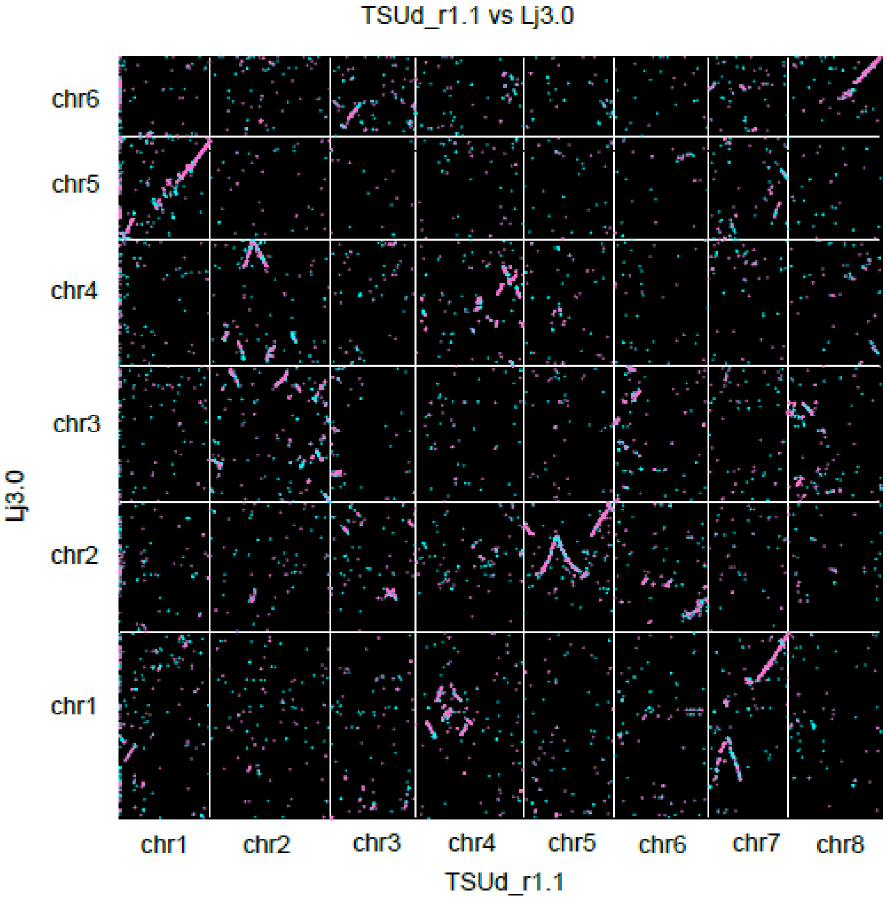

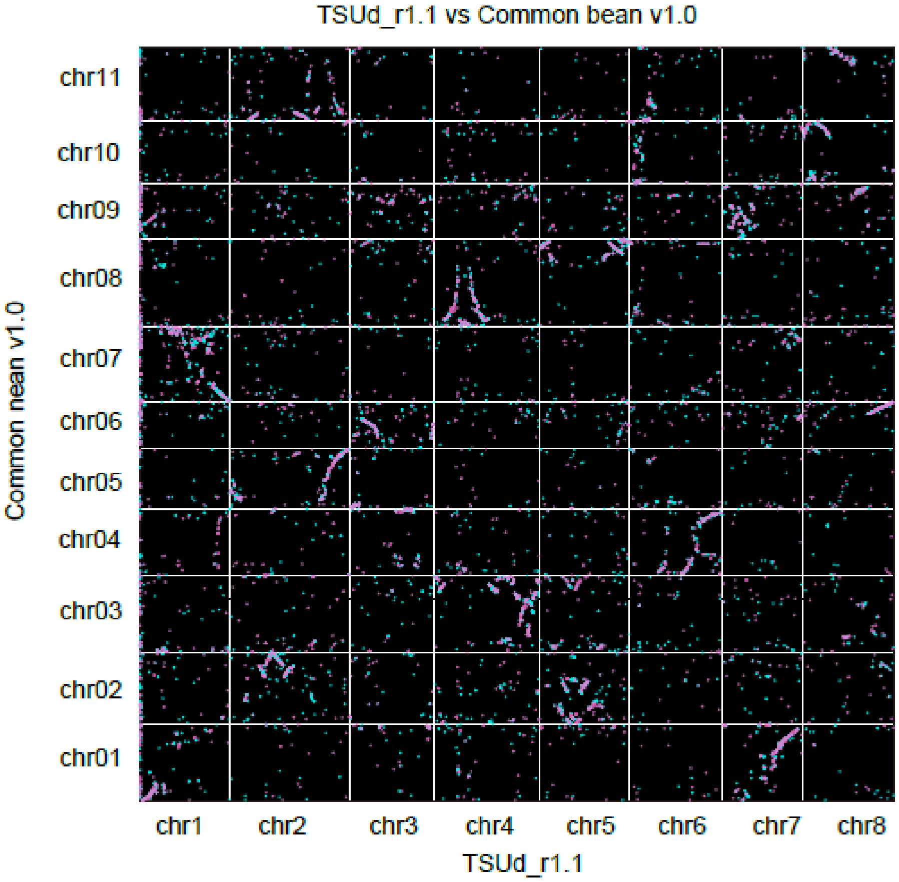

Supplementary Fig. S7. Graphical view of syntenic relationship between subterranean clover and *L. japonicus* (left) and subterranean clover and common bean (right). Pink and blue dots show homlogous sequences of Tsud_r1.1 with forward and revers direction against the reference sequences.

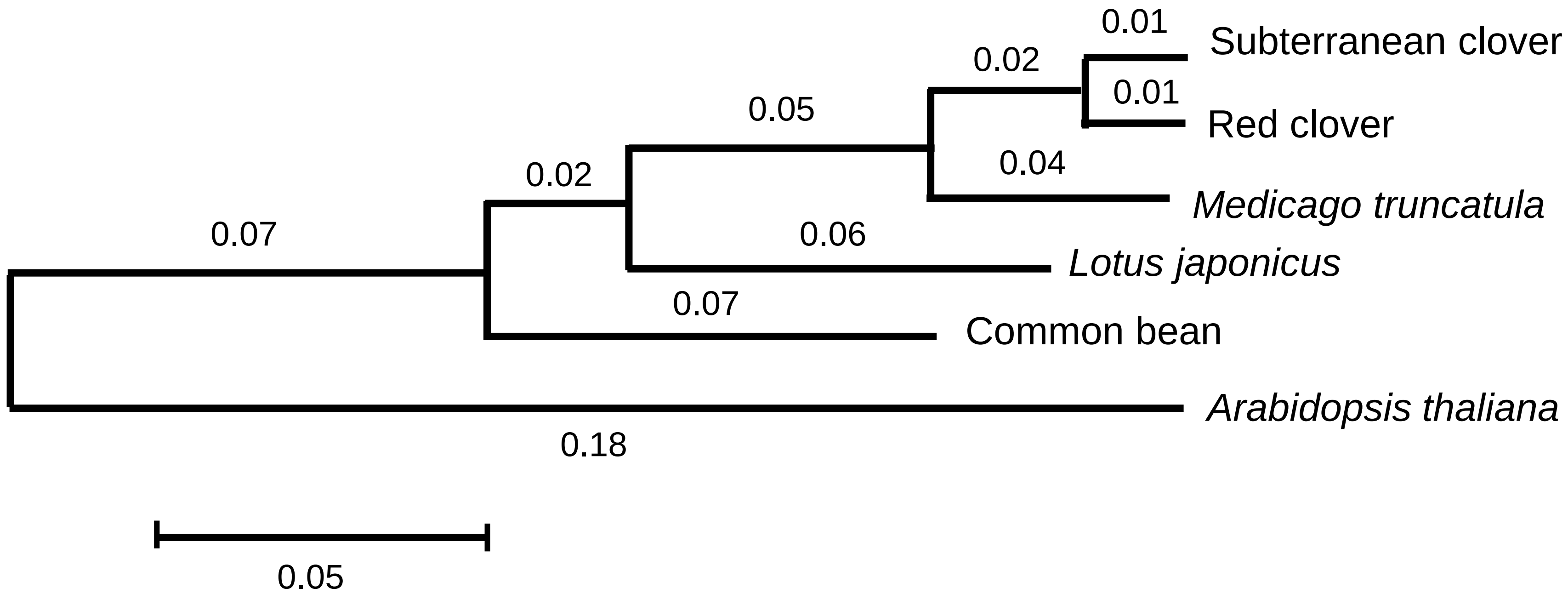

Supplementary Fig. S8. A phylogenetic tree of 280 common single copy genes of the five legume species and *A. thaliana* with genetic distances.

Supplementary Table S1. Daliak genome sequences used for *de novo* genome assembly.

Library	Expected insert size (kb)	Sequencing platforms	Read length (base)	Number of reads	Number of reads (after trimming)	Total bases (after trimming)	Accession #[a)]
Single-end	-	Roche 454	506[b)]	4,570,392	4,570,391	2,780,976,783	DRR032043
Single-end	-	Illumina Hiseq	97	55,076,339	38,399,064	3,724,709,208	DRR018263
Paired-end	270 bp	Illumina Hiseq	97	238,701,300	200,302,236	19,429,316,892	DRR018264
Paired-end	520 bp	Illumina Miseq	251	31,996,086	26,059,161	6,540,849,411	DRR032041
Paired-end	660 bp	Illumina Miseq	301	51,929,760	33,863,153	10,192,809,053	DRR032042
Mate-pair	2Kb	Illumina Hiseq	101	402,871,510	149,780,311	15,127,811,411	DRR032035
Mate-pair	5Kb	Illumina Hiseq	101	392,809,648	145,132,179	14,658,350,079	DRR032036
Mate-pair	8Kb	Illumina Hiseq	101	419,466,186	211,575,494	21,369,124,894	DRR032037
Mate-pair	10Kb	Illumina Hiseq	101	504,962,644	282,278,289	28,510,107,189	DRR032038
Mate-pair	15Kb	Illumina Hiseq	101	365,114,372	179,556,686	18,135,225,286	DRR032039
Mate-pair	20Kb	Illumina Hiseq	101	385,504,222	171,238,621	17,295,100,721	DRR032040

[a)] EXPERIMENT accession numbers registered in DDBJ Sequence Read Archive (DRA).

[b)] Average length

Supplemental Table S2. *De novo* assembly statistics.

		Newbler	SOAPdenovo	GapCloser	GAM-NGS	SSPACE(-k 3)		Draft genome
		454+MiSeq OF	Hiseq SE and PE reads (kmer=61)	Scaffold / HiSeq reads	master=Newbler , slave=GapCloser	GAM-NGS / MatePair reads	Exclude potential contaminated sequences	TSUd_r1.0
	A	137,980,096	133,067,456	141,051,289	138,165,060	139,156,750	139,097,257	138,029,660
	T	138,561,662	131,607,349	139,450,166	138,651,800	139,155,453	139,096,383	138,029,637
	G	69,027,292	66,214,164	70,190,930	69,122,335	69,632,813	69,596,809	69,027,352
	C	69,212,078	66,890,216	70,924,618	69,307,980	69,568,160	69,532,699	68,962,939
	n	188	0	0	0	7,695	7,695	7,695
	N	0	18,019,994	133,296	5,186	58,186,325	58,176,630	58,176,614
	others	0	0	0	0	0	0	0
	Total	414,781,316	415,799,179	421,750,299	415,252,361	475,707,196	475,507,473	472,233,897
	GC%	33.30%	32.00%	33.50%	33.30%	29.30%	29.30%	29.20%
	Total(ACGT)	414,781,128	397,779,185	421,617,003	415,247,175	417,513,176	417,323,148	414,049,588
	GC%(ACGT)	33.30%	33.50%	33.50%	33.30%	33.30%	33.30%	33.30%
	Number of contigs	101,010	603,937	603,937	92,279	44,900	44,332	27,228
	Total length of contigs	414,781,316	415,799,179	421,750,299	415,252,361	475,707,196	475,507,473	472,233,897
	Average length of contigs	4,106	688	698	4,499	10,594	10,726	17,343
	Max length of contigs	167,045	81,505	82,275	216,636	4,704,020	4,704,020	4,704,020
	Min length of contigs	100	100	100	100	100	100	300
	N50 length	13,363	6,388	6,380	17,057	319,420	319,420	322,970
100bp>=	Number of contigs	101,010	603,937	603,937	92,279	44,900	44,332	27,228
	Total length of contigs	414,781,316	415,799,179	421,750,299	415,252,361	475,707,196	475,507,473	472,233,897
	Average length of contigs	4,106	688	698	4,499	10,594	10,726	17,343
150bp>=	Number of contigs	94,334	190,095	190,095	85,613	39,935	39,468	27,228
	Total length of contigs	413,957,879	366,283,325	372,234,445	414,430,175	475,090,236	474,903,107	472,233,897
	Average length of contigs	4,388	1,926	1,958	4,840	11,896	12,032	17,343
200bp>=	Number of contigs	89,127	141,408	141,400	80,431	35,079	34,707	27,228
	Total length of contigs	413,054,710	357,953,677	363,903,292	413,531,396	474,244,796	474,074,206	472,233,897
	Average length of contigs	4,634	2,531	2,573	5,141	13,519	13,659	17,343
300bp>=	Number of contigs	81,366	114,086	113,350	72,775	27,446	27,228	27,228
	Total length of contigs	411,140,963	351,432,544	357,200,390	411,644,576	472,366,715	472,233,897	472,233,897
	Average length of contigs	5,052	3,080	3,151	5,656	17,210	17,343	17,343
400bp>=	Number of contigs	75,670	94,754	97,587	67,279	22,128	21,983	21,983
	Total length of contigs	409,161,689	344,766,029	351,753,101	409,735,552	470,523,968	470,416,422	470,416,422
	Average length of contigs	5,407	3,638	3,604	6,090	21,263	21,399	21,399
500bp>=	Number of contigs	70,852	85,034	87,352	62,742	17,948	17,857	17,857
	Total length of contigs	407,001,667	340,413,831	347,177,142	407,701,559	468,655,431	468,572,183	468,572,183
	Average length of contigs	5,744	4,003	3,974	6,498	26,111	26,240	26,240
1Kbp>=	Number of contigs	54,878	58,414	60,214	48,129	7,806	7,788	7,788
	Total length of contigs	395,472,153	321,677,828	327,966,600	397,180,582	461,600,733	461,565,830	461,565,830
	Average length of contigs	7,206	5,506	5,446	8,252	59,134	59,266	59,266
2Kbp>=	Number of contigs	39,179	39,675	40,489	34,360	4,374	4,369	4,369
	Total length of contigs	372,929,356	295,179,563	300,055,418	377,470,839	457,128,422	457,109,265	457,109,265
	Average length of contigs	9,518	7,439	7,410	10,985	104,510	104,625	104,625
3Kbp>=	Number of contigs	30,768	30,610	31,097	27,224	3,745	3,742	3,742
	Total length of contigs	352,314,739	272,906,771	276,973,937	360,009,073	455,593,178	455,578,584	455,578,584
	Average length of contigs	11,450	8,915	8,906	13,223	121,653	121,747	121,747
4Kbp>=	Number of contigs	25,298	24,742	25,118	22,691	3,495	3,492	3,492
	Total length of contigs	333,365,833	252,573,260	256,253,300	344,312,038	454,744,070	454,729,476	454,729,476
	Average length of contigs	13,177	10,208	10,201	15,173	130,112	130,220	130,220
5Kbp>=	Number of contigs	21,375	20,422	20,700	19,437	3,373	3,372	3,372
	Total length of contigs	315,830,075	233,241,931	236,505,632	329,781,284	454,201,577	454,196,068	454,196,068
	Average length of contigs	14,775	11,421	11,425	16,966	134,658	134,696	134,696

Supplementary Table S3. Statistics of a SNP linkage map and numbers of anchored scaffolds.

	Linnkage map					Number of ahcnored scaffolds (TSUd_r1.1).
	Number of mapped SNPs	Number of Bins	Length (cM)	Mean distance between SNPs (cM)	Mean distance between bins (cM)	
Chr1	2929	240	243.9	0.083	1.02	152
Chr2	6281	330	301.8	0.048	0.91	266
Chr3	3306	291	282.8	0.086	0.97	217
Chr4	6439	303	269.0	0.042	0.89	250
Chr5	4888	268	255.9	0.052	0.95	215
Chr6	3886	258	270.6	0.070	1.05	240
Chr7	3775	221	217.0	0.057	0.98	134
Chr8	3897	242	243.1	0.062	1.00	228
Total	35,341	2,153	2,084	0.059	0.97	1,702

Supplementary Table S4. Assembly statistics of TSUd_r1.1 and pseudomolecules.

	TSUd_r1.1	TSUd_pseudo mol Chr1-8	TSUd_r1.1_pseudomolecule.fasta (Chr1~8 + scaffold)	TSUw_r1.0[a)]
A	138,019,896	115,916,616	138,022,334	22,103,280
T	138,039,401	115,951,722	138,036,963	22,087,679
G	69,026,825	57,228,356	69,026,313	11,798,469
C	68,963,466	57,157,876	68,963,978	11,805,590
n	7,695	6,246	7,695	1,449
N	57,776,905	54,887,320	74,716,905	19,829,585
others	0	0	0	0
Total	471,834,188	401,148,136	488,774,188	87,626,052
Total (ATGC)	414,049,588	346,254,570	414,049,588	67,795,018
GC% (ATGC)	33.3	33.0	33.3	34.8
Number of sequences	27,424	8	25,730	25,722
Total length of sequences (bp)	471,834,188	401,148,136	488,774,188	87,626,052
Numbers of included TSUd_r1.1 scaffolds	-	-	-	25,722
gap (N10000)	-	-	-	-
Scaffolds length	-	-	-	-

	Tsud_chr1	Tsud_chr2	Tsud_chr3	Tsud_chr4	Tsud_chr5	Tsud_chr6	Tsud_chr7	Tsud_chr8	Chr0	TSUw_r1.0[a)]
A	14,250,573	18,446,754	12,584,179	16,429,415	13,794,029	13,858,257	12,790,090	13,763,319	22,103,280	22,103,280
T	14,260,745	18,448,151	12,593,086	16,432,700	13,829,846	13,864,097	12,761,479	13,761,618	22,087,679	22,087,679
G	6,949,028	9,104,009	6,309,862	8,089,551	6,767,494	6,953,707	6,250,397	6,804,308	11,798,469	11,798,469
C	6,913,734	9,086,605	6,313,709	8,078,335	6,772,676	6,911,819	6,248,069	6,832,929	11,805,590	11,805,590
n	616	1,004	835	820	739	876	549	807	1,449	1,449
N	5,271,063	8,645,101	7,064,334	7,406,356	6,556,804	7,964,949	4,607,700	7,371,013	19,829,585	19,829,585
others	0	0	0	0	0	0	0	0	0	0
Total	47,645,759	63,731,624	44,866,005	56,437,177	47,721,588	49,553,705	42,658,284	48,533,994	87,626,052	87,626,052
Total (ATGC)	42,374,080	55,085,519	37,800,836	49,030,001	41,164,045	41,587,880	38,050,035	41,162,174	67,795,018	67,795,018
GC% (ATGC)	32.7	33.0	33.4	33.0	32.9	33.3	32.8	33.1	34.8	34.8
Number of sequences	1	1	1	1	1	1	1	1	25,722	25,722
Total length of sequences (bp)	47,645,759	63,731,624	44,866,005	56,437,177	47,721,588	49,553,705	42,658,284	48,533,994	87,626,052	87,626,052
Numbers of included TSUd_r1.1 scaffolds	152	266	217	250	215	240	134	228	25,722	25,722
gap (N10000)	1,510,000	2,650,000	2,160,000	2,490,000	2,140,000	2,390,000	1,330,000	2,270,000	-	-
Scaffolds length	46,135,759	61,081,624	42,706,005	53,947,177	45,581,588	47,163,705	41,328,284	46,263,994	-	-

[a)] The assembled genome sequences of Woogenellup. Assembly was done by SOAPdenovo2.

Supplementary Table S5. Length and ratio of repetitive sequences.

					TSUd_r1.1		TSUd_chr1		TSUd_chr2		TSUd_chr3		TSUd_chr4	
					Length occupied (bp)	% of whole genome[b)]	Length occupied (bp)	% of whole genome[b)]	Length occupied (bp)	% of whole core candidate genome[b)]	Length occupied (bp)	% of whole core candidate genome[b)]	Length occupied (bp)	% of whole line-specific genome[b)]
Known repeats	Interspersed repeats	Class I	SINEs		289,531	0.1	39,889	0.1	38,356	0.1	23,684	0.1	42,493	0.1
			LINEs		8,375,876	1.7	884,570	1.9	1,144,210	1.8	812,757	1.8	971,606	1.7
			LTR elements	Total	43,951,472	9.0	2,597,863	5.5	4,538,855	7.1	4,161,991	9.3	3,849,403	6.8
				Copia	26,421,465	5.4	1,670,314	3.5	2,984,859	4.7	2,664,725	5.9	2,485,444	4.4
				Gypsy	17,167,590	3.5	896,215	1.9	1,519,517	2.4	1,455,428	3.2	1,324,537	2.3
		Class II	DNA elements		8,679,302	1.8	984,309	2.1	1,189,473	1.9	770,760	1.7	1,063,126	1.9
		Unclassified			183	0.0	0	0.0	133	0.0	0	0.0	0	0.0
	Helitrons				1,234,098	0.3	51,351	0.1	168,634	0.3	149,060	0.3	88,949	0.2
	Low complexity[a)]				2,241,716	0.5	233,574	0.5	296,764	0.5	200,258	0.4	275,323	0.5
	Simple repeat				7,740,338	1.6	791,804	1.7	1,024,424	1.6	688,735	1.5	924,186	1.6
	Unknown				13,108	0.0	2,529	0.0	4,401	0.0	563	0.0	1,372	0.0
	Subtotal				72,916,494	14.9	5,619,314	11.8	8,442,750	13.2	6,852,403	15.3	7,257,660	12.9
Unique repeats	Unknown				143,707,770	29.4	11,817,328	24.8	17,077,270	26.8	13,694,818	30.5	15,020,491	26.6
	Simple repeat				191,559	0.0	18,886	0.0	25,714	0.0	16,267	0.0	22,324	0.0
	Subtotal				143,899,329	29.4	11,836,214	24.8	17,102,984	26.8	13,711,085	30.6	15,042,815	26.7
Total					216,815,823	44.4	17,455,528	36.6	25,545,734	40.1	20,563,488	45.8	22,300,475	39.5

					TSUd_chr5		TSUd_chr6		TSUd_chr7		TSUd_chr8		Chr0	
					Length occupied (bp)	% of whole line-specific genome[b)]	Length occupied (bp)	% of whole line-specific genome[b)]	Length occupied (bp)	% of whole line-specific genome[b)]	Length occupied (bp)	% of whole line-specific genome[b)]	Length occupied (bp)	% of whole line-specific genome[b)]
Known repeats	Interspersed repeats	Class I	SINEs		32,580	0.1	28,139	0.1	31,582	0.1	30,497	0.1	22,311	0.0
			LINEs		853,708	1.8	942,373	1.9	757,412	1.8	835,538	1.7	1,173,702	1.3
			LTR elements	Total	3,419,607	7.2	4,736,591	9.6	2,354,540	5.5	3,999,727	8.2	14,292,895	16.3
				Copia	2,220,268	4.7	3,133,481	6.3	1,536,942	3.6	2,502,206	5.2	7,223,226	8.2
				Gypsy	1,166,462	2.4	1,566,943	3.2	792,640	1.9	1,467,098	3.0	6,978,750	8.0
		Class II	DNA elements		876,264	1.8	848,467	1.7	840,359	2.0	854,884	1.8	1,251,660	1.4
		Unclassified			0	0.0	0	0.0	50	0.0	0	0.0	0	0.0
	Helitrons				136,654	0.3	209,165	0.4	76,104	0.2	183,565	0.4	170,616	0.2
	Low complexity[a)]				216,030	0.5	224,034	0.5	202,810	0.5	217,202	0.4	375,721	0.4
	Simple repeat				770,438	1.6	781,088	1.6	712,766	1.7	788,750	1.6	1,258,147	1.4
	Unknown				823	0.0	989	0.0	1,041	0.0	492	0.0	898	0.0
	Subtotal				6,340,276	13.3	7,810,120	15.8	5,002,967	11.7	6,943,787	14.3	18,647,217	21.3
Unique repeats	Unknown				12,864,716	27.0	14,990,411	30.3	10,379,513	24.3	14,255,651	29.4	33,607,572	38.4
	Simple repeat				19,407	0.0	17,870	0.0	18,191	0.0	19,554	0.0	33,346	0.0
	Subtotal				12,884,123	27.0	15,008,281	30.3	10,397,704	24.4	14,275,205	29.4	33,640,918	38.4
Total					19,224,399	40.3	22,818,401	46.0	15,400,671	36.1	21,218,992	43.7	52,288,135	59.7

a) Primarily poly-purine/poly-pyrimidine stretches, or regions of extremely high AT or GC content. Stretches of DNA (100 bp) were masked when they were >87% AT or >89% GC, and 30 bp stretches were masked when they contained 29 A/T (or GC) nucleotides.

b) N bases were excluded from the calculation.

Supplementary Table S6. Length and ratio of unique repetitive sequences in the higher ratio of repetitive sequences was observed in the subterranean clover pseudomolecules[a)].

	Common[b)]		subterranean clover specific		Total
	length (bp)	(%)	length (bp)	(%)	length (bp)
Chr1	7,904,210	68.5	3,631,815	31.5	11,536,025
Chr2	10,490,020	63.1	6,142,585	36.9	16,632,605
Chr3	7,600,585	57.1	5,701,902	42.9	13,302,487
Chr4	9,414,513	64.3	5,229,790	35.7	14,644,303
Chr5	8,081,505	64.4	4,468,044	35.6	12,549,549
Chr6	8,561,755	58.8	6,006,949	41.2	14,568,704
Chr7	6,984,383	68.9	3,147,948	31.1	10,132,331
Chr8	8,341,880	60.2	5,523,449	39.8	13,865,329
Total	67,378,851	62.8	39,852,482	37.2	107,231,333

[a)] Exclude simple repeats and orverlapped repeat sequences.

[b)]Commonly observed in at least one of the other four legume species (red clover v2.1, *M. truncatula* 4.0v1, *L. japonicus* rel.3.0 and common bean v1.0)

Supplementary Table S7. Simple sequence repeat (SSR) distribution in the assembled genomes.

SSR pattern	*Trifolium subterraneum* (TSUd_r1.1_pseudomolecule)			*Trifolium pratense* L. (v2.1)			*Medicago truncatula* (4.0v1)			*Lotus japonicus* (Lj3.0)			*Phaseolus vulgaris* (v1.0)		
	Number of SSR in genome assembly	Frequency (Number/100 kb)		Number of SSR in genome assembly	Frequency (Number/100 kb)		Number of SSR in genome assembly	Frequency (Number/100 kb)		Number of SSR in genome assembly	Frequency (Number/100 kb)		Number of SSR in genome assembly	Frequency (Number/100 kb)	
		Overall	CDS		Overall	CDS		Overall	CDS		Overall	CDS		Overall	CDS
AC	2,451	0.50	0.01	2,055	0.59	0.05	2,599	0.63	0.03	2,125	0.47	0.03	1,319	0.25	0.02
AG	6,339	1.30	0.08	4,423	1.28	0.34	6,546	1.59	0.23	6,622	1.48	0.23	3,565	0.68	0.14
AT	17,482	3.58	0.00	12,303	3.56	0.11	16,074	3.90	0.02	11,445	2.56	0.02	25,713	4.93	0.01
CG	17	0.00	-	4	0.00	-	18	0.00	-	11	0.00	-	8	0.00	-
Sub-total	26,289	5.38	0.09	18,785	5.43	0.50	25,237	6.12	0.28	20,203	4.51	0.28	30,605	5.86	0.17
AAC	2,034	0.42	0.79	1,642	0.47	1.18	2,638	0.64	0.77	1,627	0.36	0.77	496	0.10	0.33
AAG	3,059	0.63	1.05	2,567	0.74	1.80	3,028	0.74	1.63	4,239	0.95	1.51	2,451	0.47	1.33
AAT	13,860	2.84	0.24	8,799	2.54	0.66	5,663	1.38	0.18	3,339	0.75	0.03	11,143	2.14	0.09
ACC	757	0.15	0.85	637	0.18	0.79	666	0.16	0.71	3,231	0.72	1.65	562	0.11	0.87
ACG	36	0.01	0.03	32	0.01	0.05	54	0.01	0.06	64	0.01	0.07	44	0.01	0.09
ACT	414	0.08	0.12	524	0.15	0.20	244	0.06	0.10	233	0.05	0.06	114	0.02	0.02
AGC	193	0.04	0.22	246	0.07	0.39	229	0.06	0.31	380	0.08	0.57	363	0.07	0.38
AGG	324	0.07	0.42	371	0.11	0.55	430	0.10	0.48	1,134	0.25	0.83	350	0.07	0.71
ATC	2,159	0.44	1.13	1,866	0.54	1.74	1,517	0.37	1.17	1,723	0.39	1.09	990	0.19	0.70
CCG	34	0.01	0.03	13	0.00	0.02	28	0.01	0.03	244	0.05	0.26	113	0.02	0.29
Sub-total	22,870	4.69	4.88	16,697	4.81	7.38	14,497	3.53	5.44	16,214	3.61	6.84	16,626	3.20	4.81
AAAC	398	0.08	0.00	573	0.17	0.02	309	0.08	0.00	287	0.06	0.00	171	0.03	-
AAAG	505	0.10	0.01	619	0.18	0.05	660	0.16	0.02	664	0.15	0.02	516	0.10	0.01
AAAT	4,803	0.98	-	4,271	1.23	0.03	2,858	0.69	0.02	1,591	0.36	0.00	4,822	0.93	0.00
AACC	54	0.01	-	81	0.02	0.01	31	0.01	0.00	52	0.01	0.01	33	0.01	-
AACG	7	0.00	-	12	0.00	-	7	0.00	-	15	0.00	-	8	0.00	-
AACT	545	0.11	-	489	0.14	0.00	201	0.05	-	306	0.07	-	63	0.01	-
AAGC	20	0.00	-	44	0.01	0.00	31	0.01	0.01	26	0.01	-	25	0.00	-
AAGG	30	0.01	0.00	58	0.02	0.01	70	0.02	0.01	60	0.01	-	56	0.01	-
AAGT	28	0.01	-	63	0.02	-	19	0.00	-	13	0.00	-	20	0.00	-
AATC	127	0.03	-	139	0.04	0.01	104	0.03	-	165	0.04	-	51	0.01	0.00
AATG	472	0.10	0.00	588	0.17	0.03	270	0.07	0.00	219	0.05	0.01	195	0.04	-
AATT	851	0.17	-	880	0.25	-	524	0.13	-	383	0.09	-	666	0.13	-
ACAG	7	0.00	0.00	16	0.00	0.00	25	0.01	-	22	0.00	0.00	3	0.00	-
ACCC	20	0.00	-	13	0.00	0.00	29	0.01	0.01	22	0.00	-	18	0.00	-
ACCG	1	0.00	-	1	0.00	-	-	-	-	-	-	-	49	0.01	-
ACCT	16	0.00	-	24	0.01	0.00	8	0.00	-	7	0.00	-	13	0.00	-
ACGC	42	0.01	-	102	0.03	0.00	26	0.01	-	21	0.00	0.00	9	0.00	-
ACGG	-	-	-	3	0.00	-	3	0.00	-	4	0.00	-	2	0.00	-
ACGT	1	0.00	-	5	0.00	-	4	0.00	-	1	0.00	-	3	0.00	-
ACTC	119	0.02	-	178	0.05	0.02	113	0.03	0.01	227	0.05	0.01	129	0.02	0.00
ACTG	9	0.00	-	10	0.00	-	3	0.00	-	5	0.00	-	5	0.00	-
AGCC	6	0.00	0.00	11	0.00	-	5	0.00	0.00	7	0.00	-	3	0.00	-
AGCG	4	0.00	-	5	0.00	0.00	6	0.00	-	14	0.00	-	1	0.00	-
AGCT	29	0.01	-	57	0.02	-	30	0.01	-	49	0.01	-	10	0.00	-
AGGC	7	0.00	-	9	0.00	-	1	0.00	-	4	0.00	-	1	0.00	-
AGGG	151	0.03	0.01	69	0.02	0.01	203	0.05	0.01	84	0.02	0.01	24	0.00	0.01
ATAC	1,400	0.29	0.00	344	0.10	0.01	315	0.08	-	176	0.04	0.01	177	0.03	-
ATAG	232	0.05	0.00	404	0.12	0.00	400	0.10	0.01	913	0.20	0.01	254	0.05	-
ATCC	38	0.01	-	70	0.02	0.01	36	0.01	-	19	0.00	-	33	0.01	-
ATCG	3	0.00	-	6	0.00	0.00	6	0.00	-	7	0.00	-	1	0.00	-
ATGC	38	0.01	0.00	44	0.01	0.01	64	0.02	0.00	22	0.00	-	60	0.01	-
CCCG	-	-	-	-	-	-	-	-	-	-	-	-	1	0.00	-
CCGG	-	-	-	1	0.00	-	-	-	-	-	-	-	1	0.00	-
Sub-total	9,963	2.03	0.02	9,189	2.63	0.22	6,361	1.58	0.10	5,385	1.17	0.08	7,423	1.40	0.02

AAAAC	106	0.02	-	155	0.04	0.01	125	0.03	0.00	82	0.02	-	41	0.01	-
AAAAG	83	0.02	-	108	0.03	0.00	98	0.02	0.01	120	0.03	0.01	147	0.03	-
AAAAT	612	0.13	-	568	0.16	0.00	805	0.20	0.00	284	0.06	-	834	0.16	-
AAACC	115	0.02	-	86	0.02	-	119	0.03	0.00	50	0.01	0.00	33	0.01	-
AAACG	-	-	-	1	0.00	-	-	-	-	2	0.00	-	8	0.00	-
AAACT	11	0.00	-	11	0.00	-	10	0.00	-	10	0.00	-	1	0.00	-
AAAGC	4	0.00	-	8	0.00	-	1	0.00	-	6	0.00	-	3	0.00	-
AAAGG	6	0.00	-	9	0.00	-	10	0.00	-	20	0.00	-	12	0.00	0.00
AAAGT	2	0.00	-	5	0.00	-	3	0.00	-	6	0.00	-	3	0.00	-
AAATC	25	0.01	-	26	0.01	-	39	0.01	-	44	0.01	-	39	0.01	-
AAATG	14	0.00	-	26	0.01	-	25	0.01	-	25	0.01	-	15	0.00	-
AAATT	98	0.02	-	96	0.03	-	91	0.02	-	86	0.02	-	158	0.03	-
AACAC	23	0.00	-	85	0.02	0.01	49	0.01	-	56	0.01	-	39	0.01	0.00
AACAG	8	0.00	-	12	0.00	-	15	0.00	-	25	0.01	-	8	0.00	-
AACAT	21	0.00	-	36	0.01	-	37	0.01	0.00	7	0.00	-	4	0.00	-
AACCC	8	0.00	-	4	0.00	-	12	0.00	-	44	0.01	0.00	7	0.00	-
AACCG	8	0.00	-	6	0.00	-	23	0.01	-	50	0.01	-	5	0.00	-
AACCT	5	0.00	-	3	0.00	-	3	0.00	-	10	0.00	-	3	0.00	-
AACGC	1	0.00	-	4	0.00	-	3	0.00	-	1	0.00	-	-	-	-
AACGG	-	-	-	1	0.00	-	1	0.00	-	7	0.00	-	-	-	-
AACGT	-	-	-	-	-	-	1	0.00	-	1	0.00	-	1	0.00	-
AACTC	3	0.00	-	12	0.00	0.00	12	0.00	-	21	0.00	-	5	0.00	-
AACTG	4	0.00	-	4	0.00	0.00	5	0.00	-	6	0.00	-	2	0.00	-
AACTT	3	0.00	-	1	0.00	-	2	0.00	-	-	-	-	-	-	-
AAGAC	2	0.00	-	3	0.00	-	2	0.00	-	3	0.00	-	4	0.00	-
AAGAG	24	0.00	-	48	0.01	0.00	56	0.01	0.01	94	0.02	0.01	106	0.02	0.01
AAGAT	9	0.00	-	17	0.00	-	24	0.01	-	11	0.00	-	6	0.00	-
AAGCC	-	-	-	3	0.00	0.00	3	0.00	-	2	0.00	-	1	0.00	-
AAGCG	-	-	-	-	-	-	1	0.00	-	-	-	-	-	-	-
AAGCT	-	-	-	4	0.00	-	-	-	-	6	0.00	-	1	0.00	-
AAGGC	-	-	-	2	0.00	-	1	0.00	-	1	0.00	-	1	0.00	-
AAGGG	1	0.00	-	3	0.00	-	11	0.00	-	16	0.00	0.00	6	0.00	-
AAGGT	3	0.00	-	2	0.00	-	-	-	-	3	0.00	-	4	0.00	-
AAGTC	-	-	-	2	0.00	-	2	0.00	-	1	0.00	-	1	0.00	-
AAGTG	4	0.00	-	4	0.00	-	5	0.00	-	12	0.00	-	10	0.00	-
AATAC	25	0.01	-	20	0.01	-	25	0.01	0.00	13	0.00	-	5	0.00	-
AATAG	9	0.00	-	26	0.01	-	14	0.00	-	13	0.00	-	8	0.00	-
AATAT	125	0.03	-	80	0.02	-	107	0.03	-	163	0.04	-	191	0.04	-
AATCC	8	0.00	-	18	0.01	0.00	12	0.00	-	14	0.00	-	2	0.00	-
AATCG	3	0.00	-	2	0.00	-	4	0.00	-	7	0.00	-	2	0.00	-
AATCT	17	0.00	-	50	0.01	-	13	0.00	-	18	0.00	-	4	0.00	-
AATGC	7	0.00	-	31	0.01	0.00	10	0.00	-	6	0.00	-	4	0.00	-
AATGG	12	0.00	-	25	0.01	-	16	0.00	0.00	14	0.00	-	3	0.00	-
AATGT	10	0.00	-	24	0.01	-	16	0.00	-	8	0.00	-	4	0.00	-
AATTC	26	0.01	-	46	0.01	0.01	44	0.01	-	43	0.01	0.00	12	0.00	-
ACACC	5	0.00	-	7	0.00	-	10	0.00	-	26	0.01	-	9	0.00	-
ACACG	-	-	-	3	0.00	-	1	0.00	-	3	0.00	-	-	-	-
ACACT	5	0.00	-	9	0.00	0.00	2	0.00	-	4	0.00	-	2	0.00	-
ACAGC	2	0.00	-	5	0.00	-	2	0.00	-	11	0.00	-	5	0.00	-
ACAGG	-	-	-	1	0.00	-	1	0.00	-	2	0.00	-	1	0.00	-
ACAGT	-	-	-	3	0.00	-	1	0.00	-	2	0.00	-	-	-	-
ACCAG	2	0.00	-	-	-	-	2	0.00	-	-	-	-	2	0.00	-
ACCCC	6	0.00	-	-	-	-	8	0.00	-	10	0.00	-	2	0.00	-
ACCCG	-	-	-	2	0.00	-	-	-	-	51	0.01	-	5	0.00	-
ACCCT	1	0.00	-	4	0.00	-	2	0.00	-	-	-	-	-	-	-
ACCGC	-	-	-	-	-	-	1	0.00	-	1	0.00	-	1	0.00	-

ACCGG	2	0.00	-	4	0.00	-	2	0.00	-	6	0.00	-	-	-	-
ACCGT	-	-	-	-	-	-	1	0.00	-	-	-	-	-	-	-
ACCTC	3	0.00	-	4	0.00	-	3	0.00	-	12	0.00	0.00	1	0.00	-
ACCTG	1	0.00	-	1	0.00	-	1	0.00	-	3	0.00	-	-	-	-
ACGAG	-	-	-	2	0.00	-	1	0.00	-	2	0.00	-	-	-	-
ACGCC	-	-	-	-	-	-	3	0.00	-	5	0.00	-	2	0.00	-
ACGCT	-	-	-	2	0.00	-	-	-	-	-	-	-	-	-	-
ACGGG	1	0.00	-	-	-	-	-	-	-	-	-	-	-	-	-
ACTAG	3	0.00	-	8	0.00	-	3	0.00	-	1	0.00	-	2	0.00	-
ACTCC	2	0.00	0.00	2	0.00	-	1	0.00	-	28	0.01	0.00	-	-	-
ACTCG	2	0.00	-	-	-	-	1	0.00	-	1	0.00	-	-	-	-
ACTCT	-	-	-	4	0.00	-	-	-	-	2	0.00	-	2	0.00	-
ACTGC	-	-	-	6	0.00	-	1	0.00	-	1	0.00	-	-	-	-
AGAGC	1	0.00	-	4	0.00	-	-	-	-	2	0.00	-	1	0.00	-
AGAGG	7	0.00	-	7	0.00	-	14	0.00	0.00	14	0.00	0.00	5	0.00	-
AGCCC	2	0.00	-	1	0.00	-	-	-	-	5	0.00	-	3	0.00	-
AGCCG	-	-	-	-	-	-	-	-	-	11	0.00	-	1	0.00	-
AGCCT	-	-	-	1	0.00	-	1	0.00	-	-	-	-	1	0.00	-
AGCGG	-	-	-	-	-	-	-	-	-	1	0.00	-	-	-	-
AGCTC	-	-	-	3	0.00	-	-	-	-	-	-	-	-	-	-
AGGCC	-	-	-	71	0.02	-	35	0.01	0.00	7	0.00	-	-	-	-
AGGCG	-	-	-	-	-	-	-	-	-	2	0.00	-	2	0.00	-
AGGGC	-	-	-	-	-	-	-	-	-	1	0.00	-	-	-	-
AGGGG	83	0.02	-	63	0.02	-	95	0.02	0.01	129	0.03	-	4	0.00	-
ATACC	3	0.00	-	5	0.00	-	2	0.00	-	5	0.00	-	-	-	-
ATACG	5	0.00	-	-	-	-	3	0.00	-	1	0.00	-	-	-	-
ATACT	13	0.00	-	34	0.01	-	16	0.00	-	10	0.00	-	3	0.00	-
ATAGC	2	0.00	-	16	0.00	0.00	3	0.00	-	6	0.00	-	7	0.00	-
ATAGG	3	0.00	-	25	0.01	-	5	0.00	-	9	0.00	0.00	1	0.00	-
ATATC	26	0.01	-	38	0.01	-	32	0.01	-	39	0.01	-	17	0.00	-
ATCAC	3	0.00	-	14	0.00	-	5	0.00	-	9	0.00	-	5	0.00	-
ATCAG	1	0.00	-	3	0.00	-	2	0.00	-	3	0.00	-	-	-	-
ATCCC	1	0.00	-	10	0.00	-	3	0.00	-	1	0.00	-	3	0.00	-
ATCCG	1	0.00	-	-	-	-	2	0.00	-	3	0.00	-	1	0.00	-
ATCGC	-	-	-	-	-	-	1	0.00	-	3	0.00	-	-	-	-
ATCTC	4	0.00	-	24	0.01	0.00	19	0.00	-	17	0.00	-	16	0.00	-
ATGAC	4	0.00	-	5	0.00	-	5	0.00	-	5	0.00	-	3	0.00	-
ATGCC	1	0.00	-	7	0.00	-	4	0.00	-	8	0.00	0.00	3	0.00	-
CCCCG	7	0.00	0.00	-	-	-	2	0.00	-	2	0.00	-	11	0.00	-
CCCGG	-	-	-	1	0.00	-	1	0.00	-	1	0.00	-	-	-	-
CCGCG	-	-	-	-	-	-	-	-	-	1	0.00	-	-	-	-
Sub-total	1,647	0.30	0.00	2,076	0.52	0.03	2,152	0.46	0.03	1,877	0.34	0.02	1,864	0.32	0.01

AAAAAC	24	0.00	0.00	44	0.01	0.00	40	0.01	0.01	33	0.01	-	10	0.00	-
AAAAAG	34	0.01	-	27	0.01	0.00	46	0.01	0.01	32	0.01	-	73	0.01	-
AAAAAT	113	0.02	-	104	0.03	0.00	130	0.03	-	86	0.02	-	122	0.02	-
AAAACC	9	0.00	-	6	0.00	-	12	0.00	0.01	11	0.00	0.02	5	0.00	0.00
AAAACG	1	0.00	-	-	-	-	5	0.00	-	1	0.00	-	13	0.00	-
AAAACT	15	0.00	-	5	0.00	-	1	0.00	-	1	0.00	-	1	0.00	-
AAAAGC	2	0.00	-	2	0.00	0.00	2	0.00	0.00	4	0.00	-	1	0.00	-
AAAAGG	5	0.00	0.01	1	0.00	-	5	0.00	0.00	8	0.00	0.00	5	0.00	0.00
AAAAGT	2	0.00	-	1	0.00	-	3	0.00	-	-	-	-	-	-	-
AAAATC	23	0.00	0.00	29	0.01	0.01	26	0.01	0.01	23	0.01	0.00	9	0.00	-
AAAATG	6	0.00	-	13	0.00	0.00	18	0.00	-	7	0.00	-	11	0.00	-
AAAATT	15	0.00	-	13	0.00	-	20	0.00	-	24	0.01	-	41	0.01	-
AAACAC	7	0.00	-	12	0.00	0.01	14	0.00	0.00	19	0.00	-	7	0.00	0.01
AAACAG	4	0.00	-	2	0.00	0.00	8	0.00	0.00	3	0.00	-	4	0.00	0.00
AAACAT	3	0.00	-	1	0.00	-	9	0.00	-	4	0.00	-	5	0.00	-
AAACCC	15	0.00	0.01	52	0.02	0.00	3	0.00	0.00	45	0.01	0.01	4	0.00	0.00
AAACCT	-	-	-	-	-	-	6	0.00	0.00	3	0.00	0.00	1	0.00	-
AAACGC	-	-	-	-	-	-	3	0.00	0.00	1	0.00	-	-	-	-
AAACGG	-	-	-	-	-	-	-	-	-	1	0.00	0.00	1	0.00	-
AAACTC	2	0.00	-	2	0.00	-	1	0.00	-	3	0.00	-	3	0.00	-
AAACTG	1	0.00	0.00	2	0.00	-	5	0.00	0.00	9	0.00	0.00	1	0.00	-
AAACTT	-	-	-	1	0.00	-	-	-	-	-	-	-	-	-	-
AAAGAC	1	0.00	-	-	-	-	1	0.00	-	-	-	-	-	-	-
AAAGAG	6	0.00	-	7	0.00	0.00	14	0.00	0.00	17	0.00	0.00	54	0.01	0.03
AAAGAT	14	0.00	-	15	0.00	-	5	0.00	0.00	4	0.00	-	3	0.00	-
AAAGCC	-	-	-	1	0.00	0.00	3	0.00	-	1	0.00	0.00	1	0.00	0.00
AAAGCG	-	-	-	-	-	-	1	0.00	-	-	-	-	-	-	-
AAAGCT	-	-	-	-	-	-	1	0.00	-	-	-	-	-	-	-
AAAGGC	-	-	-	-	-	-	1	0.00	-	3	0.00	-	2	0.00	-
AAAGGG	1	0.00	-	-	-	-	3	0.00	0.00	4	0.00	0.00	3	0.00	0.00
AAAGGT	-	-	-	-	-	-	1	0.00	0.00	-	-	-	-	-	-
AAAGTC	-	-	-	2	0.00	-	-	-	-	1	0.00	-	96	0.02	-
AAAGTG	-	-	-	1	0.00	-	3	0.00	0.00	4	0.00	-	-	-	-
AAAGTT	-	-	-	1	0.00	-	-	-	-	-	-	-	-	-	-
AAATAC	4	0.00	-	-	-	-	1	0.00	-	1	0.00	-	6	0.00	-
AAATAG	2	0.00	-	7	0.00	0.00	4	0.00	-	2	0.00	0.00	1	0.00	-
AAATAT	14	0.00	-	14	0.00	-	19	0.00	-	19	0.00	-	44	0.01	-
AAATCC	8	0.00	-	6	0.00	0.00	3	0.00	-	2	0.00	-	4	0.00	-
AAATCG	-	-	-	-	-	-	1	0.00	-	2	0.00	-	-	-	-
AAATCT	2	0.00	-	2	0.00	-	2	0.00	0.00	6	0.00	-	1	0.00	-
AAATGC	4	0.00	-	2	0.00	-	3	0.00	-	1	0.00	-	-	-	-
AAATGG	2	0.00	0.00	6	0.00	0.00	10	0.00	0.01	13	0.00	0.01	5	0.00	-
AAATGT	2	0.00	-	-	-	-	-	-	-	1	0.00	-	2	0.00	-
AAATTC	8	0.00	0.00	19	0.01	0.00	10	0.00	0.01	18	0.00	0.01	11	0.00	0.00
AAATTG	5	0.00	-	12	0.00	-	40	0.01	0.00	27	0.01	-	11	0.00	-
AAATTT	2	0.00	-	-	-	-	2	0.00	-	9	0.00	-	7	0.00	-
AACAAG	5	0.00	0.01	10	0.00	0.01	8	0.00	0.01	10	0.00	0.01	3	0.00	-
AACACC	5	0.00	0.01	6	0.00	0.01	4	0.00	0.00	8	0.00	0.01	13	0.00	0.01
AACACG	-	-	-	-	-	-	2	0.00	0.00	2	0.00	0.00	-	-	-
AACACT	-	-	-	1	0.00	-	-	-	-	3	0.00	-	2	0.00	-
AACAGC	4	0.00	0.01	5	0.00	0.01	1	0.00	0.00	15	0.00	0.03	1	0.00	0.00
AACAGG	1	0.00	-	-	-	-	1	0.00	0.00	1	0.00	-	-	-	-
AACAGT	1	0.00	-	1	0.00	0.00	1	0.00	0.00	2	0.00	-	-	-	-
AACATC	-	-	-	4	0.00	0.00	9	0.00	0.02	8	0.00	0.01	4	0.00	0.01
AACATG	3	0.00	0.00	3	0.00	-	5	0.00	0.01	8	0.00	0.01	5	0.00	0.01
AACCAC	13	0.00	0.01	2	0.00	0.00	2	0.00	0.00	5	0.00	-	4	0.00	0.01
AACCAG	4	0.00	0.01	6	0.00	0.02	3	0.00	0.01	15	0.00	0.02	3	0.00	0.01
AACCAT	1	0.00	0.00	4	0.00	-	1	0.00	-	1	0.00	0.00	1	0.00	0.00

AACCCC	-	-	-	-	-	-	7	0.00	-	13	0.00	0.00	1	0.00	0.00
AACCCG	1	0.00	-	-	-	-	1	0.00	0.00	10	0.00	0.00	5	0.00	0.01
AACCCT	4	0.00	0.01	2	0.00	0.01	8	0.00	0.01	8	0.00	0.01	15	0.00	0.01
AACCGC	-	-	-	1	0.00	-	1	0.00	-	-	-	-	-	-	-
AACCGT	-	-	-	-	-	-	11	0.00	-	1	0.00	0.00	-	-	-
AACCTC	-	-	-	2	0.00	0.00	-	-	-	11	0.00	0.01	-	-	-
AACCTG	-	-	-	-	-	-	-	-	-	2	0.00	-	-	-	-
AACCTT	-	-	-	2	0.00	-	-	-	-	1	0.00	-	1	0.00	-
AACGAC	-	-	-	1	0.00	-	2	0.00	-	-	-	-	1	0.00	-
AACGAG	-	-	-	-	-	-	-	-	-	4	0.00	-	1	0.00	0.00
AACGAT	-	-	-	-	-	-	-	-	-	-	-	-	2	0.00	-
AACGCC	-	-	-	-	-	-	-	-	-	2	0.00	0.01	-	-	-
AACGGC	-	-	-	-	-	-	-	-	-	2	0.00	-	1	0.00	-
AACGGT	-	-	-	-	-	-	-	-	-	-	-	-	6	0.00	-
AACGTG	-	-	-	1	0.00	0.00	-	-	-	-	-	-	-	-	-
AACTAC	-	-	-	1	0.00	0.00	1	0.00	0.00	1	0.00	-	1	0.00	-
AACTAG	2	0.00	-	2	0.00	-	1	0.00	-	3	0.00	-	-	-	-
AACTAT	1	0.00	-	1	0.00	-	1	0.00	-	1	0.00	-	1	0.00	-
AACTCC	1	0.00	0.00	1	0.00	0.00	2	0.00	-	4	0.00	0.00	2	0.00	0.01
AACTCG	-	-	-	-	-	-	-	-	-	-	-	-	1	0.00	0.00
AACTCT	-	-	-	1	0.00	-	1	0.00	-	-	-	-	1	0.00	-
AACTGC	-	-	-	1	0.00	-	-	-	-	12	0.00	0.01	-	-	-
AACTGG	-	-	-	3	0.00	0.00	2	0.00	0.00	4	0.00	0.01	-	-	-
AACTGT	1	0.00	-	4	0.00	0.00	1	0.00	-	4	0.00	0.01	1	0.00	-
AACTTC	-	-	-	3	0.00	0.00	3	0.00	0.00	4	0.00	0.00	3	0.00	-
AACTTG	1	0.00	-	3	0.00	0.00	2	0.00	-	2	0.00	-	3	0.00	0.00
AAGACC	-	-	-	-	-	-	1	0.00	0.00	-	-	-	2	0.00	-
AAGACG	-	-	-	1	0.00	0.00	1	0.00	0.00	1	0.00	-	3	0.00	0.00
AAGAGC	4	0.00	0.00	1	0.00	-	1	0.00	0.00	1	0.00	-	-	-	-
AAGAGG	9	0.00	0.01	3	0.00	0.00	24	0.01	0.02	36	0.01	0.03	14	0.00	0.02
AAGATC	-	-	-	1	0.00	0.00	1	0.00	0.00	2	0.00	-	-	-	-
AAGATG	5	0.00	-	10	0.00	0.01	24	0.01	0.03	79	0.02	0.06	13	0.00	0.02
AAGCAC	1	0.00	0.00	-	-	-	2	0.00	-	4	0.00	0.00	-	-	-
AAGCAG	2	0.00	-	3	0.00	0.00	3	0.00	0.00	9	0.00	0.01	1	0.00	-
AAGCAT	-	-	-	-	-	-	1	0.00	-	2	0.00	-	1	0.00	-
AAGCCC	5	0.00	0.00	2	0.00	0.00	1	0.00	0.00	4	0.00	0.00	154	0.03	0.01
AAGCCG	-	-	-	-	-	-	-	-	-	1	0.00	0.00	1	0.00	0.00
AAGCCT	-	-	-	1	0.00	-	-	-	-	-	-	-	-	-	-
AAGCGG	-	-	-	-	-	-	-	-	-	1	0.00	-	-	-	-
AAGCTC	-	-	-	1	0.00	-	3	0.00	-	3	0.00	0.00	2	0.00	0.00
AAGCTG	-	-	-	-	-	-	-	-	-	2	0.00	-	-	-	-
AAGGAC	1	0.00	-	-	-	-	1	0.00	0.00	2	0.00	0.00	-	-	-
AAGGAG	3	0.00	0.00	3	0.00	-	10	0.00	0.02	53	0.01	0.02	11	0.00	0.02
AAGGAT	-	-	-	1	0.00	-	-	-	-	1	0.00	0.00	4	0.00	0.01
AAGGCG	-	-	-	-	-	-	1	0.00	-	-	-	-	-	-	-
AAGGCT	-	-	-	-	-	-	1	0.00	0.00	-	-	-	-	-	-
AAGGGG	-	-	-	1	0.00	-	-	-	-	6	0.00	-	1	0.00	-
AAGGGT	-	-	-	-	-	-	1	0.00	-	1	0.00	-	1	0.00	-
AAGGTC	1	0.00	0.00	1	0.00	0.00	-	-	-	-	-	-	1	0.00	-
AAGGTG	1	0.00	0.00	2	0.00	-	8	0.00	0.01	12	0.00	0.02	3	0.00	0.01
AAGTAG	-	-	-	2	0.00	0.00	4	0.00	0.01	1	0.00	-	3	0.00	-
AAGTAT	-	-	-	1	0.00	-	-	-	-	-	-	-	-	-	-
AAGTCC	-	-	-	1	0.00	0.00	-	-	-	-	-	-	-	-	-
AAGTGC	1	0.00	-	2	0.00	0.00	4	0.00	-	4	0.00	-	1	0.00	0.00
AAGTGG	-	-	-	2	0.00	0.00	4	0.00	0.01	3	0.00	-	2	0.00	-
AAGTGT	-	-	-	-	-	-	1	0.00	-	-	-	-	-	-	-
AATAAC	6	0.00	0.00	11	0.00	0.00	9	0.00	0.01	1	0.00	-	27	0.01	-
AATAAG	3	0.00	-	4	0.00	-	5	0.00	-	-	-	-	3	0.00	-
AATACC	1	0.00	0.00	-	-	-	1	0.00	0.00	4	0.00	0.01	-	-	-
AATACG	-	-	-	1	0.00	-	-	-	-	-	-	-	-	-	-

AATACT	1	0.00	-	2	0.00	-	-	-	-	-	-	-	3	0.00	-
AATAGC	2	0.00	-	4	0.00	-	2	0.00	-	5	0.00	0.00	2	0.00	0.00
AATAGG	-	-	-	4	0.00	-	6	0.00	-	2	0.00	-	13	0.00	-
AATAGT	4	0.00	0.00	6	0.00	0.00	8	0.00	0.00	4	0.00	-	2	0.00	-
AATATC	1	0.00	-	5	0.00	-	3	0.00	0.00	181	0.04	-	-	-	-
AATATG	1	0.00	-	2	0.00	0.00	8	0.00	0.00	13	0.00	0.01	5	0.00	0.00
AATATT	4	0.00	-	6	0.00	-	3	0.00	-	10	0.00	-	47	0.01	-
AATCAC	5	0.00	0.00	10	0.00	0.01	5	0.00	0.00	13	0.00	0.01	3	0.00	0.00
AATCAG	4	0.00	0.00	7	0.00	0.01	7	0.00	0.01	7	0.00	0.00	4	0.00	0.01
AATCAT	16	0.00	0.01	17	0.00	0.01	13	0.00	0.00	4	0.00	-	9	0.00	-
AATCCC	-	-	-	2	0.00	-	4	0.00	0.00	13	0.00	-	3	0.00	0.00
AATCCG	-	-	-	-	-	-	1	0.00	-	2	0.00	-	1	0.00	-
AATCCT	3	0.00	0.00	10	0.00	0.00	-	-	-	2	0.00	-	1	0.00	-
AATCGC	1	0.00	-	-	-	-	-	-	-	3	0.00	0.00	1	0.00	-
AATCGG	-	-	-	1	0.00	-	1	0.00	0.00	10	0.00	0.01	2	0.00	-
AATCGT	-	-	-	-	-	-	-	-	-	1	0.00	-	-	-	-
AATCTC	1	0.00	-	5	0.00	0.00	6	0.00	0.01	9	0.00	0.00	2	0.00	-
AATCTG	-	-	-	1	0.00	-	2	0.00	0.00	10	0.00	0.00	1	0.00	0.00
AATCTT	6	0.00	-	17	0.00	-	2	0.00	-	4	0.00	-	2	0.00	-
AATGAC	1	0.00	-	2	0.00	-	4	0.00	0.00	2	0.00	-	2	0.00	-
AATGAG	1	0.00	-	4	0.00	0.00	6	0.00	0.00	8	0.00	0.00	5	0.00	-
AATGAT	4	0.00	-	8	0.00	-	7	0.00	0.00	7	0.00	0.00	3	0.00	-
AATGCC	-	-	-	2	0.00	-	2	0.00	0.00	4	0.00	-	-	-	-
AATGCG	-	-	-	-	-	-	1	0.00	-	2	0.00	-	-	-	-
AATGCT	2	0.00	0.00	6	0.00	0.00	1	0.00	-	5	0.00	-	-	-	-
AATGGC	-	-	-	6	0.00	0.00	1	0.00	-	3	0.00	0.01	1	0.00	-
AATGGG	1	0.00	-	2	0.00	0.00	-	-	-	8	0.00	0.00	2	0.00	0.01
AATGGT	1	0.00	-	7	0.00	0.01	4	0.00	0.00	3	0.00	0.00	-	-	-
AATGTC	-	-	-	-	-	-	1	0.00	-	-	-	-	-	-	-
AATGTG	1	0.00	-	4	0.00	-	8	0.00	0.00	7	0.00	-	2	0.00	0.00
AATGTT	4	0.00	0.00	-	-	-	2	0.00	-	1	0.00	-	5	0.00	-
AATTAC	3	0.00	-	6	0.00	-	2	0.00	0.00	2	0.00	-	1	0.00	-
AATTAG	1	0.00	-	5	0.00	0.00	3	0.00	-	9	0.00	-	2	0.00	-
AATTAT	1	0.00	-	7	0.00	0.00	4	0.00	-	3	0.00	-	10	0.00	-
AATTCC	1	0.00	-	3	0.00	-	6	0.00	0.00	9	0.00	0.01	3	0.00	0.01
AATTGC	-	-	-	1	0.00	-	-	-	-	1	0.00	-	1	0.00	-
ACACAG	1	0.00	-	-	-	-	1	0.00	-	5	0.00	-	1	0.00	-
ACACCC	-	-	-	1	0.00	-	1	0.00	0.00	7	0.00	0.00	3	0.00	-
ACACCG	-	-	-	-	-	-	1	0.00	-	4	0.00	0.00	1	0.00	-
ACACCT	22	0.00	-	1	0.00	-	-	-	-	3	0.00	0.00	-	-	-
ACACGC	-	-	-	1	0.00	0.00	-	-	-	7	0.00	-	4	0.00	-
ACACGG	-	-	-	2	0.00	-	1	0.00	-	-	-	-	2	0.00	-
ACACTC	-	-	-	5	0.00	0.00	3	0.00	-	11	0.00	0.01	4	0.00	-
ACACTG	-	-	-	2	0.00	-	1	0.00	-	-	-	-	-	-	-
ACAGAG	-	-	-	-	-	-	3	0.00	0.00	5	0.00	-	3	0.00	0.00
ACAGCC	-	-	-	1	0.00	0.00	1	0.00	0.00	-	-	-	3	0.00	0.01
ACAGGC	-	-	-	2	0.00	0.00	-	-	-	2	0.00	0.01	-	-	-
ACAGGG	-	-	-	1	0.00	0.00	-	-	-	-	-	-	1	0.00	0.00
ACAGGT	-	-	-	-	-	-	1	0.00	-	-	-	-	-	-	-
ACAGTC	1	0.00	-	2	0.00	-	-	-	-	1	0.00	-	-	-	-
ACAGTG	-	-	-	1	0.00	-	-	-	-	4	0.00	-	2	0.00	0.00
ACCACG	-	-	-	-	-	-	1	0.00	-	3	0.00	-	2	0.00	0.01
ACCAGC	2	0.00	0.00	3	0.00	0.00	-	-	-	12	0.00	0.01	2	0.00	0.01
ACCAGG	-	-	-	-	-	-	2	0.00	-	1	0.00	-	-	-	-
ACCCAG	-	-	-	-	-	-	-	-	-	2	0.00	-	1	0.00	0.00
ACCCCC	2	0.00	-	-	-	-	1	0.00	-	5	0.00	0.00	-	-	-
ACCCGG	-	-	-	-	-	-	-	-	-	-	-	-	1	0.00	-
ACCCGT	-	-	-	-	-	-	-	-	-	1	0.00	-	-	-	-
ACCCTC	-	-	-	-	-	-	1	0.00	0.00	24	0.01	0.01	11	0.00	0.00
ACCCTG	-	-	-	-	-	-	-	-	-	1	0.00	0.00	-	-	-

ACCGCG	-	-	-	1	0.00	-	-	-	-	-	-	-	-	-	-
ACCGCT	-	-	-	-	-	-	-	-	-	1	0.00	-	-	-	-
ACCGGC	-	-	-	2	0.00	0.00	-	-	-	2	0.00	0.00	2	0.00	0.01
ACCGGT	-	-	-	1	0.00	-	-	-	-	-	-	-	-	-	-
ACCGTC	-	-	-	1	0.00	-	-	-	-	-	-	-	-	-	-
ACCTCC	1	0.00	0.00	1	0.00	0.00	4	0.00	0.01	20	0.00	0.01	2	0.00	0.01
ACCTCG	-	-	-	-	-	-	-	-	-	1	0.00	-	-	-	-
ACCTCT	-	-	-	-	-	-	-	-	-	4	0.00	0.00	-	-	-
ACCTGC	1	0.00	0.00	-	-	-	1	0.00	0.00	2	0.00	0.00	3	0.00	-
ACCTGG	-	-	-	-	-	-	-	-	-	2	0.00	0.00	2	0.00	0.00
ACGAGC	-	-	-	-	-	-	-	-	-	2	0.00	-	-	-	-
ACGAGG	-	-	-	1	0.00	0.00	2	0.00	0.00	-	-	-	-	-	-
ACGCCC	-	-	-	-	-	-	-	-	-	7	0.00	-	-	-	-
ACGCCG	-	-	-	-	-	-	-	-	-	1	0.00	0.00	-	-	-
ACGCGC	-	-	-	-	-	-	-	-	-	1	0.00	-	-	-	-
ACGGAG	-	-	-	-	-	-	-	-	-	4	0.00	-	-	-	-
ACGGCC	-	-	-	-	-	-	-	-	-	-	-	-	1	0.00	-
ACGGCG	-	-	-	-	-	-	-	-	-	5	0.00	0.01	1	0.00	0.00
ACGGGC	-	-	-	-	-	-	1	0.00	-	3	0.00	0.00	-	-	-
ACGGGG	-	-	-	-	-	-	1	0.00	0.00	-	-	-	-	-	-
ACTACC	-	-	-	-	-	-	1	0.00	-	2	0.00	0.00	-	-	-
ACTAGC	-	-	-	-	-	-	1	0.00	0.00	4	0.00	0.00	-	-	-
ACTAGG	1	0.00	-	-	-	-	-	-	-	1	0.00	-	-	-	-
ACTAGT	-	-	-	4	0.00	0.00	-	-	-	2	0.00	-	-	-	-
ACTCAG	-	-	-	1	0.00	0.00	3	0.00	0.01	4	0.00	-	1	0.00	0.01
ACTCCC	-	-	-	-	-	-	1	0.00	0.00	-	-	-	2	0.00	0.00
ACTCCG	-	-	-	-	-	-	1	0.00	0.00	5	0.00	0.00	2	0.00	-
ACTCCT	-	-	-	2	0.00	-	-	-	-	1	0.00	-	1	0.00	-
ACTCGG	-	-	-	-	-	-	-	-	-	-	-	-	1	0.00	0.00
ACTCTC	6	0.00	0.00	3	0.00	0.00	9	0.00	-	45	0.01	0.01	4	0.00	0.01
ACTCTG	-	-	-	-	-	-	2	0.00	0.00	-	-	-	-	-	-
ACTGAG	-	-	-	1	0.00	0.00	1	0.00	0.00	6	0.00	0.01	1	0.00	-
ACTGCC	-	-	-	1	0.00	-	-	-	-	5	0.00	0.00	-	-	-
ACTGCG	-	-	-	-	-	-	1	0.00	0.00	2	0.00	0.00	1	0.00	-
ACTGCT	2	0.00	0.00	-	-	-	1	0.00	-	8	0.00	0.00	-	-	-
ACTGGC	-	-	-	1	0.00	0.00	-	-	-	2	0.00	0.00	-	-	-
ACTGGT	-	-	-	-	-	-	-	-	-	3	0.00	0.00	-	-	-
AGAGCC	1	0.00	-	-	-	-	-	-	-	6	0.00	0.01	1	0.00	0.00
AGAGCG	-	-	-	-	-	-	-	-	-	6	0.00	-	-	-	-
AGAGCT	-	-	-	1	0.00	-	-	-	-	-	-	-	-	-	-
AGAGGC	-	-	-	1	0.00	-	1	0.00	-	5	0.00	0.00	1	0.00	0.00
AGAGGG	17	0.00	-	5	0.00	0.00	29	0.01	0.00	15	0.00	0.01	3	0.00	0.00
AGCAGG	1	0.00	-	-	-	-	5	0.00	0.01	4	0.00	0.00	-	-	-
AGCCCC	-	-	-	-	-	-	-	-	-	2	0.00	0.00	-	-	-
AGCCCG	-	-	-	-	-	-	-	-	-	-	-	-	1	0.00	-
AGCCGC	-	-	-	-	-	-	1	0.00	0.00	-	-	-	-	-	-
AGCCTC	1	0.00	0.00	1	0.00	-	2	0.00	-	6	0.00	0.00	-	-	-
AGCCTG	-	-	-	1	0.00	0.00	1	0.00	-	5	0.00	0.01	2	0.00	0.01
AGCGGC	-	-	-	-	-	-	1	0.00	-	-	-	-	-	-	-
AGCGGG	1	0.00	-	1	0.00	0.00	-	-	-	-	-	-	-	-	-
AGCTCC	2	0.00	0.00	1	0.00	-	1	0.00	0.00	3	0.00	0.00	1	0.00	0.00
AGGCCC	-	-	-	1	0.00	0.00	-	-	-	2	0.00	-	1	0.00	0.00
AGGCGG	-	-	-	-	-	-	1	0.00	0.00	2	0.00	0.00	-	-	-
AGGGCG	-	-	-	-	-	-	-	-	-	1	0.00	-	-	-	-
AGGGGC	-	-	-	-	-	-	2	0.00	-	-	-	-	3	0.00	0.01
AGGGGG	2	0.00	-	-	-	-	2	0.00	0.00	2	0.00	-	1	0.00	-
ATACAC	1	0.00	-	3	0.00	-	2	0.00	-	7	0.00	-	1	0.00	-
ATACAG	-	-	-	-	-	-	-	-	-	1	0.00	-	-	-	-

ATACCC	-	-	-	2	0.00	0.00	2	0.00	-	-	-	-	-	-	-
ATACCG	1	0.00	-	-	-	-	-	-	-	-	-	-	-	-	-
ATACGT	-	-	-	-	-	-	-	-	-	-	-	-	1	0.00	-
ATACTC	-	-	-	-	-	-	-	-	-	2	0.00	-	-	-	-
ATACTG	-	-	-	-	-	-	-	-	-	2	0.00	-	-	-	-
ATAGAC	-	-	-	1	0.00	0.00	2	0.00	-	2	0.00	-	1	0.00	-
ATAGAG	3	0.00	0.00	8	0.00	0.00	17	0.00	-	4	0.00	-	4	0.00	0.01
ATAGCC	2	0.00	-	3	0.00	-	1	0.00	0.00	1	0.00	-	-	-	-
ATAGGC	1	0.00	-	-	-	-	-	-	-	3	0.00	-	-	-	-
ATAGGG	-	-	-	-	-	-	1	0.00	0.00	-	-	-	3	0.00	0.00
ATAGTC	-	-	-	1	0.00	-	1	0.00	-	1	0.00	-	-	-	-
ATAGTG	4	0.00	0.00	2	0.00	-	3	0.00	0.00	1	0.00	-	-	-	-
ATATAC	2	0.00	-	13	0.00	-	11	0.00	-	6	0.00	-	13	0.00	-
ATATAG	23	0.00	-	36	0.01	-	7	0.00	-	72	0.02	-	8	0.00	0.00
ATATCC	-	-	-	3	0.00	-	-	-	-	7	0.00	-	-	-	-
ATCACC	2	0.00	0.00	7	0.00	0.01	11	0.00	0.01	21	0.00	0.03	5	0.00	0.00
ATCACG	-	-	-	1	0.00	0.00	1	0.00	0.00	6	0.00	0.00	1	0.00	0.00
ATCAGC	-	-	-	-	-	-	2	0.00	-	9	0.00	0.00	3	0.00	0.01
ATCAGG	-	-	-	-	-	-	-	-	-	-	-	-	1	0.00	0.00
ATCATG	2	0.00	-	1	0.00	0.00	1	0.00	0.00	1	0.00	-	-	-	-
ATCCAC	1	0.00	0.00	1	0.00	0.00	2	0.00	0.00	5	0.00	0.00	3	0.00	0.00
ATCCCC	-	-	-	-	-	-	1	0.00	-	-	-	-	1	0.00	-
ATCCGC	-	-	-	1	0.00	-	-	-	-	2	0.00	-	1	0.00	0.00
ATCCTC	2	0.00	0.00	3	0.00	0.00	5	0.00	0.01	19	0.00	0.02	3	0.00	0.00
ATCGAC	1	0.00	-	-	-	-	-	-	-	-	-	-	1	0.00	-
ATCGCC	1	0.00	-	-	-	-	1	0.00	-	2	0.00	-	-	-	-
ATCGGC	1	0.00	0.00	-	-	-	-	-	-	2	0.00	0.00	-	-	-
ATCGTC	-	-	-	2	0.00	0.00	2	0.00	0.00	1	0.00	0.00	-	-	-
ATCTAC	-	-	-	1	0.00	-	-	-	-	5	0.00	-	2	0.00	-
ATCTCC	-	-	-	4	0.00	0.00	5	0.00	0.00	13	0.00	0.02	4	0.00	0.00
ATCTCG	-	-	-	-	-	-	-	-	-	1	0.00	-	-	-	-
ATCTGC	1	0.00	-	3	0.00	0.00	1	0.00	-	3	0.00	0.00	-	-	-
ATCTGG	-	-	-	-	-	-	-	-	-	2	0.00	-	-	-	-
ATGACC	3	0.00	0.01	2	0.00	0.00	2	0.00	0.00	1	0.00	-	3	0.00	0.01
ATGAGC	-	-	-	1	0.00	0.00	1	0.00	-	3	0.00	-	1	0.00	-
ATGCCC	-	-	-	-	-	-	-	-	-	3	0.00	-	1	0.00	-
ATGGCC	-	-	-	-	-	-	-	-	-	1	0.00	-	-	-	-
ATGTAC	-	-	-	-	-	-	3	0.00	-	-	-	-	-	-	-
ATGTCC	1	0.00	-	2	0.00	-	3	0.00	0.00	-	-	-	-	-	-
ATGTGC	-	-	-	1	0.00	-	2	0.00	-	2	0.00	-	1	0.00	-
CCCCCG	-	-	-	-	-	-	1	0.00	-	-	-	-	-	-	-
CCGGCG	-	-	-	-	-	-	-	-	-	1	0.00	0.00	-	-	-
Sub-total	633	0.03	0.11	857	0.10	0.14	988	0.10	0.30	1,749	0.20	0.59	1,178	0.13	0.36
Total	61,402	12.43	5.10	47,604	13.49	8.27	49,235	11.79	6.15	45,428	9.83	7.81	57,696	10.91	5.37

Supplemental Table S8. Statistics of candidate genes predicted by MAKER and Augustus 3.0.2.

		MAKER	Augustus	Integrated
Total	Number of sequences	28,372	14,334	42,706
	Total length (bp)	26,446,191	21,518,826	47,965,017
	Average length (bp)	932	1,501	1,123
	Max length (bp)	14,295	15,417	15,417
	Min length (bp)	150	153	150
	N50 length (bp)	1,299	2,073	1,548
	A	7,911,463	6,470,083	14,381,546
	T	7,552,592	6,067,898	13,620,490
	G	6,021,713	4,975,944	10,997,657
	C	4,958,722	3,997,181	8,955,903
	N	1,701	7,720	9,421
	Total (ATGC)	26,444,490	21,511,106	47,955,596
	GC% (GC/ATGC)	41.5	41.7	41.6
≥500 bp	Number of sequences	18,459	12,238	30,697
	Total length (bp)	23,330,754	20,762,572	44,093,326
	Average length (bp)	1,263	1,696	1,436
≥1 kb	Number of sequences	9,809	8,184	17,993
	Total length (bp)	17,026,347	17,768,297	34,794,644
	Average length (bp)	1,735	2,171	1,933
≥5 kb	Number of sequences	106	221	327
	Total length (bp)	663,291	1,312,777	1,976,068
	Average length (bp)	6,257	5,940	6,043

Supplementary Table S9. Numbers of putative tRNA and rRNA encoding genes identified in TSUd_r1.1.

tRNA

Encode gene	*Trifolium subterraneum* (TSUd_r1.1)	Red clover (v2.1)	*M. truncatula* (4.0v1)	*L. japonicus* (Lj3.0)	Common bean (v1.0)
Ala	52	59	58	40	41
Arg	56	53	49	54	43
Asn	36	31	51	28	22
Asp	47	35	43	32	30
Cys	22	26	18	76	16
Gln	25	26	27	23	21
Glu	40	45	37	40	28
Gly	65	61	63	48	51
His	19	20	24	19	15
Ile	40	48	36	29	30
Leu	84	71	72	57	56
Lys	55	48	46	41	43
Met	49	45	62	48	39
Phe	27	30	27	22	28
Pro	40	40	46	46	48
Ser	63	62	60	44	50
Thr	39	40	39	36	28
Trp	20	21	22	21	16
Tyr	27	26	19	22	17
Val	44	43	49	34	41
Subtotal	850	830	848	760	663
Pseudo[a)]	148	72	207	80	12
SeC[a)]	1	0	1	12	1
Sup[a)]	3	4	10	0	1
Undet[a)]	5	3	8	6	4
Total	1007	909	1074	858	681

rRNA

Encode gene	*Trifolium subterraneum* (TSUd_r1.1)	Red clover (v2.1)	*M. truncatula* (4.0v1)	*L. japonicus* (Lj3.0)	Common bean (v1.0)
5.8S rRNA	5	6	24	6	8
18S rRNA	37	28	40	27	48
25S rRNA	50	48	87	63	83
Total	92	82	151	96	139

[a)] Pseudo, SeC, Sup, and Undet represent pseudogenes, selenocysteine tRNAs, possible suppressor tRNAs, and tRNAs with undetermined/unknown isotypes, respectively.

[b)] Accession numbers used for identification or 5.8S, 18S, and 25S rRNAs were X52320.1, X16077.1, and X52320.1, respectively.

Supplementary Table S10. Number of putative genes encoding enzymes those mapped on KEGG pathways catagorized as '1. Metabolism'

Pathway category	Pathway name	Subterranean clover (TSUd_r1.1)	Red clover (v2.1)	*Medicago truncatula* (4.0v1)	*Lotus japonicus* (Lj3.0)	Common bean (v1.0)	Map#
1.1 Carbohydrate metabolism	Glycolysis / Gluconeogenesis	31	30	31	30	31	map00010
	Citrate cycle (TCA cycle)	20	20	20	20	19	map00020
	Pentose phosphate pathway	18	19	19	17	18	map00030
	Pentose and glucuronate interconversions	16	14	15	14	16	map00040
	Fructose and mannose metabolism	20	20	21	16	20	map00051
	Galactose metabolism	20	18	19	20	19	map00052
	Ascorbate and aldarate metabolism	15	12	16	15	15	map00053
	Starch and sucrose metabolism	37	35	35	37	35	map00500
	Amino sugar and nucleotide sugar metabolism	40	37	41	38	39	map00520
	Pyruvate metabolism	30	28	33	29	30	map00620
	Glyoxylate and dicarboxylate metabolism	25	26	27	25	27	map00630
	Propanoate metabolism	14	15	16	14	15	map00640
	Butanoate metabolism	12	12	15	13	13	map00650
	C5-Branched dibasic acid metabolism	5	5	5	5	5	map00660
	Inositol phosphate metabolism	23	22	23	21	23	map00562
1.2 Energy metabolism	Oxidative phosphorylation	67	72	88	73	77	map00190
	Photosynthesis	26	28	48	38	39	map00195
	Photosynthesis - antenna proteins	9	11	12	8	12	map00196
	Carbon fixation in photosynthetic organisms	23	24	26	23	25	map00710
	Carbon fixation pathways in prokaryotes	15	15	14	14	14	map00720
	Methane metabolism	19	19	19	19	19	map00680
	Nitrogen metabolism	10	12	12	12	12	map00910
	Sulfur metabolism	14	14	14	14	14	map00920
1.3 Lipid metabolism	Fatty acid biosynthesis	16	16	16	14	16	map00061
	Fatty acid elongation	9	9	9	9	10	map00062
	Fatty acid degradation	12	11	13	12	12	map00071
	Synthesis and degradation of ketone bodies	3	3	3	3	3	map00072
	Cutin, suberine and wax biosynthesis	10	11	11	11	11	map00073
	Steroid biosynthesis	15	17	17	17	17	map00100
	Primary bile acid biosynthesis	0	0	0	0	0	map00120
	Secondary bile acid biosynthesis	0	0	0	0	0	map00121
	Steroid hormone biosynthesis	6	7	7	6	6	map00140
	Glycerolipid metabolism	26	28	29	29	29	map00561
	Glycerophospholipid metabolism	36	42	42	39	43	map00564
	Ether lipid metabolism	11	11	11	11	11	map00565
	Sphingolipid metabolism	18	17	19	17	18	map00600
	Arachidonic acid metabolism	10	9	10	10	9	map00590
	Linoleic acid metabolism	6	6	6	6	6	map00591
	alpha-Linolenic acid metabolism	16	15	16	14	16	map00592
	Biosynthesis of unsaturated fatty acids	1	1	1	0	1	map01040
1.4 Nucleotide metabolism	Purine metabolism	81	90	98	82	97	map00230
	Pyrimidine metabolism	69	73	80	67	78	map00240

1.5 Amino acid metabolism	Alanine, aspartate and glutamate metabolism	24	26	28	28	28	map00250
	Glycine, serine and threonine metabolism	35	34	38	36	37	map00260
	Cysteine and methionine metabolism	34	34	36	34	36	map00270
	Valine, leucine and isoleucine degradation	21	20	21	21	21	map00280
	Valine, leucine and isoleucine biosynthesis	10	10	10	10	10	map00290
	Lysine biosynthesis	11	10	11	10	10	map00300
	Lysine degradation	14	12	13	13	14	map00310
	Arginine and proline metabolism	40	36	42	39	41	map00330
	Histidine metabolism	10	10	13	12	12	map00340
	Tyrosine metabolism	19	17	20	22	20	map00350
	Phenylalanine metabolism	20	16	22	20	19	map00360
	Tryptophan metabolism	12	12	14	12	13	map00380
	Phenylalanine, tyrosine and tryptophan biosynthesis	24	23	25	24	25	map00400
1.6 Metabolism of other amino acids	beta-Alanine metabolism	16	16	17	17	17	map00410
	Taurine and hypotaurine metabolism	3	3	3	3	3	map00430
	Phosphonate and phosphinate metabolism	4	4	4	4	4	map00440
	Selenocompound metabolism	10	10	10	9	10	map00450
	Cyanoamino acid metabolism	12	12	13	12	11	map00460
	D-Glutamine and D-glutamate metabolism	1	1	1	1	1	map00471
	D-Arginine and D-ornithine metabolism	0	0	1	1	1	map00472
	D-Alanine metabolism	0	0	1	1	0	map00473
	Glutathione metabolism	16	17	17	16	17	map00480
1.7 Glycan biosynthesis and metabolism	N-Glycan biosynthesis	31	31	32	25	31	map00510
	Various types of N-glycan biosynthesis	9	8	9	6	8	map00513
	Mucin type O-Glycan biosynthesis	1	1	1	1	1	map00512
	Other types of O-glycan biosynthesis	0	0	0	0	0	map00514
	Glycosaminoglycan biosynthesis - CS/DS	2	2	2	2	2	map00532
	Glycosaminoglycan biosynthesis - HS/Hep	3	2	3	3	3	map00534
	Glycosaminoglycan biosynthesis - KS	0	0	0	0	0	map00533
	Glycosaminoglycan degradation	5	5	5	5	5	map00531
	Glycosylphosphatidylinositol(GPI)-anchor biosynthesis	18	20	21	16	22	map00563
	Glycosphingolipid biosynthesis - lacto and neolacto series	0	0	0	0	0	map00601
	Glycosphingolipid biosynthesis - globo series	4	4	4	4	4	map00603
	Glycosphingolipid biosynthesis - ganglio series	3	3	3	3	3	map00604
	Lipopolysaccharide biosynthesis	8	8	8	8	8	map00540
	Peptidoglycan biosynthesis	2	2	3	4	2	map00550
	Other glycan degradation	0	0	0	0	0	map00511
1.8 Metabolism of cofactors and vitamins	Thiamine metabolism	10	8	9	9	10	map00730
	Riboflavin metabolism	8	10	10	11	10	map00740
	Vitamin B6 metabolism	8	8	7	8	8	map00750
	Nicotinate and nicotinamide metabolism	13	12	13	11	13	map00760
	Pantothenate and CoA biosynthesis	17	17	17	17	17	map00770
	Biotin metabolism	7	8	8	7	8	map00780
	Lipoic acid metabolism	3	3	3	3	3	map00785
	Folate biosynthesis	11	10	11	11	11	map00790
	One carbon pool by folate	13	14	13	13	12	map00670
	Retinol metabolism	9	9	9	9	9	map00830
	Porphyrin and chlorophyll metabolism	30	31	30	29	31	map00860
	Ubiquinone and other terpenoid-quinone biosynthesis	19	17	20	18	19	map00130

1.9 Metabolism of terpenoids and polyketides	Terpenoid backbone biosynthesis	27	29	30	26	30	map00900
	Monoterpenoid biosynthesis	3	5	5	5	6	map00902
	Sesquiterpenoid and triterpenoid biosynthesis	6	8	9	8	8	map00909
	Diterpenoid biosynthesis	11	12	13	11	12	map00904
	Carotenoid biosynthesis	23	22	23	21	23	map00906
	Brassinosteroid biosynthesis	4	8	8	7	8	map00905
	Insect hormone biosynthesis	1	1	1	1	1	map00981
	Zeatin biosynthesis	6	6	6	6	7	map00908
	Limonene and pinene degradation	4	4	4	4	4	map00903
	Geraniol degradation	2	2	3	2	2	map00281
	Type I polyketide structures	0	0	0	0	0	map01052
	Biosynthesis of 12-, 14- and 16-membered macrolides	0	0	0	0	0	map00522
	Biosynthesis of ansamycins	1	1	1	1	1	map01051
	Biosynthesis of type II polyketide backbone	0	0	0	0	0	map01056
	Biosynthesis of type II polyketide products	0	0	0	0	0	map01057
	Tetracycline biosynthesis	4	4	4	3	4	map00253
	Polyketide sugar unit biosynthesis	4	4	3	2	3	map00523
	Nonribosomal peptide structures	0	0	0	0	0	map01054
	Biosynthesis of siderophore group nonribosomal peptide	2	1	1	2	2	map01053
	Biosynthesis of vancomycin group antibiotics	1	1	1	1	1	map01055
1.10 Biosynthesis of other secondary metabolites	Phenylpropanoid biosynthesis	17	21	21	20	21	map00940
	Stilbenoid, diarylheptanoid and gingerol biosynthesis	6	7	6	7	7	map00945
	Flavonoid biosynthesis	14	17	16	17	17	map00941
	Flavone and flavonol biosynthesis	2	5	6	4	5	map00944
	Anthocyanin biosynthesis	5	5	6	6	7	map00942
	Isoflavonoid biosynthesis	11	11	11	11	11	map00943
	Indole alkaloid biosynthesis	3	3	3	2	3	map00901
	Isoquinoline alkaloid biosynthesis	10	10	13	14	12	map00950
	Tropane, piperidine and pyridine alkaloid biosynthesis	12	9	12	13	12	map00960
	Acridone alkaloid biosynthesis	0	0	0	0	0	map01058
	Caffeine metabolism	2	2	2	2	2	map00232
	Betalain biosynthesis	4	3	3	5	4	map00965
	Glucosinolate biosynthesis	0	0	0	0	0	map00966
	Benzoxazinoid biosynthesis	0	0	0	0	0	map00402
	Penicillin and cephalosporin biosynthesis	1	1	3	3	2	map00311
	Carbapenem biosynthesis	0	0	0	0	0	map00332
	Monobactam biosynthesis	0	0	0	0	0	map00261
	Clavulanic acid biosynthesis	0	0	0	0	0	map00331
	Streptomycin biosynthesis	7	7	7	6	6	map00521
	Butirosin and neomycin biosynthesis	1	1	1	1	1	map00524
	Puromycin biosynthesis	0	0	0	0	0	map00231
	Novobiocin biosynthesis	3	2	3	3	3	map00401
	Aflatoxin biosynthesis	1	1	1	1	1	map00254

1.11 Xenobiotics biodegradation and metabolism	Benzoate degradation	6	4	7	6	7	map00362
	Aminobenzoate degradation	7	8	9	8	7	map00627
	Fluorobenzoate degradation	1	1	2	1	2	map00364
	Chloroalkane and chloroalkene degradation	6	6	6	6	6	map00625
	Chlorocyclohexane and chlorobenzene degradation	4	4	5	4	5	map00361
	Toluene degradation	1	1	3	1	2	map00623
	Xylene degradation	1	0	2	1	1	map00622
	Nitrotoluene degradation	0	0	0	0	0	map00633
	Ethylbenzene degradation	1	1	2	1	1	map00642
	Styrene degradation	5	4	5	5	5	map00643
	Atrazine degradation	0	0	1	0	0	map00791
	Caprolactam degradation	1	1	1	1	1	map00930
	DDT degradation	1	1	1	1	1	map00351
	Bisphenol degradation	3	3	3	3	3	map00363
	Dioxin degradation	1	1	2	1	1	map00621
	Naphthalene degradation	3	3	3	3	3	map00626
	Polycyclic aromatic hydrocarbon degradation	3	3	3	3	3	map00624
	Furfural degradation	0	0	0	0	0	map00365
	Steroid degradation	0	0	0	0	0	map00984
	Metabolism of xenobiotics by cytochrome P450	6	6	6	7	6	map00980
	Drug metabolism - cytochrome P450	4	4	5	4	4	map00982
	Drug metabolism - other enzymes	12	12	12	12	12	map00983

Supplementary Table S11 Numbers of annotated SNPs by SnpEff program

Impact on gene function	Annotation	No. of SNPs	%
High impact	Splice acceptor variant & intron variant	11	0.03
High impact	Splice donor variant & intron variant	26	0.07
High impact	Start lost	15	0.04
High impact	Start lost & splice region variant	1	0
High impact	Stop gained	130	0.35
High impact	Stop gained & splice region variant	2	0.01
High impact	Stop lost	79	0.22
High impact	Stop lost & splice region variant	23	0.06
Moderate impact	Missense variant	4,840	13.18
Moderate impact	Missense variant & splice region variant	54	0.15
Modifier impact	Intergenic region	15,589	42.45
Modifier impact	Intron variant	10,518	28.64
Low impact	Initiator codon variant	1	0
Low impact	Splice region variant & intron variant	411	1.12
Low impact	Splice region variant & stop retained variant	6	0.02
Low impact	Splice region variant & synonymous variant	46	0.13
Low impact	Stop retained variant	15	0.04
Low impact	Synonymous variant	4,956	13.5
